# *Chara* — a living sister to the land plants with pivotal enzymic toolkit for mannan and xylan remodelling

**DOI:** 10.1111/ppl.14134

**Published:** 2023-12-29

**Authors:** Lenka Franková, Stephen C. Fry

**Affiliations:** ^1^ The Edinburgh Cell Wall Group, Institute of Molecular Plant Sciences The University of Edinburgh Edinburgh UK

## Abstract

Land‐plant transglycosylases ‘cut‐and‐paste’ cell‐wall polysaccharides by endo‐transglycosylation (transglycanases) and exo‐transglycosylation (transglycosidases). Such enzymes may remodel the wall, adjusting extensibility and adhesion. Charophytes have cell‐wall polysaccharides that broadly resemble, but appreciably differ from land‐plants'. We investigated whether *Chara vulgaris* has wall‐restructuring enzymes mirroring those of land‐plants.

Wall enzymes extracted from *Chara* were assayed *in vitro* for transglycosylase activities on various donor substrates — β‐(1→4)‐glucan‐based [xyloglucan and mixed‐linkage glucans (MLGs)], β‐(1→4)‐xylans and β‐(1→4)‐mannans — plus related acceptor substrates (tritium‐labelled oligosaccharides, XXXGol, Xyl_6_‐ol and Man_6_‐ol), thus 12 donor:acceptor permutations. Also, fluorescent oligosaccharides were incubated *in situ* with *Chara*, revealing endogenous enzyme action on endogenous (potentially novel) polysaccharides.

*Chara* enzymes acted on the glucan‐based polysaccharides with [^3^H]XXXGol as acceptor substrate, demonstrating ‘glucan:glucan‐type’ transglucanases. Such activities were unexpected because *Chara* lacks biochemically detectable xyloglucan and MLG. With xylans as donor and [^3^H]Xyl_6_‐ol (but not [^3^H]Man_6_‐ol) as acceptor, high trans‐β‐xylanase activity was detected. With mannans as donor and either [^3^H]Man_6_‐ol or [^3^H]Xyl_6_‐ol as acceptor, we detected high levels of both mannan:mannan homo‐trans‐β‐mannanase and mannan:xylan hetero‐trans‐β‐mannanase activity, showing that *Chara* can not only ‘cut/paste’ these hemicelluloses by homo‐transglycosylation but also hetero‐transglycosylate them, forming mannan→xylan (but not xylan→mannan) hybrid hemicelluloses. In *in‐situ* assays, *Chara* walls attached endogenous polysaccharides to exogenous sulphorhodamine‐labelled Man_6_‐ol, indicating transglycanase (possibly trans‐mannanase) action on endogenous polysaccharides.

In conclusion, cell‐wall transglycosylases, comparable to but different from those of land‐plants, pre‐dated the divergence of the Charophyceae from its sister clade (Coleochaetophyceae/Zygnematophyceae/land‐plants). Thus, the ability to ‘cut/paste’ wall polysaccharides is an evolutionarily ancient streptophytic trait.

## 1 INTRODUCTION

Numerous papers mention charophycean algae as attractive models for plant physiologists, biochemists and molecular biologists (Gylete et al., 2021, Domozych et al., 2016, Domozych and Bagdan, 2022), especially *Chara* spp. owing to their flexible anatomical structure, large size and easy manipulation. Charophytes are a group of green algae considered the closest living relatives of land plants. Indeed, charophytes plus land plants together constitute a broad taxon, the Streptophyta, which excludes all other algae. *Chara* and *Nitella* (stoneworts) are typical representatives of the class Charophyceae which among other classes (Coleochaetophyceae, Zygnematophyceae, Klebsormidiophyceae Chlorokybophyceae and Mesostigmatophyceae) constitute the charophytes (Domozych et al., 2016; McCourt et al., 2004, Nishiyama et al., 2018).

The Charophyceae were formerly considered as the closest living relatives to the land plants (Karol et al., 2001); however, more recent studies favour the Coleochaetophyceae (Finet et al., 2010; Leliaert et al., 2012) or Zygnematophyceae (Leebens‐Mack et al., 2019; Puttick et al., 2018; Timme et al., 2012; Turmel et al., 2006; Nishiyama et al., 2018; Domozych & LoRicco, 2023), especially the latter. Nevertheless, the Charophyceae (including *Chara* and *Nitella*) exhibit many features found in land plants, such as plasmodesmata, phragmoplast, branching, cellulose synthase rosettes, auxin signalling, apical cell growth etc. (Nishiyama et al., 2018; Foissner &Wastenys, 2014). On the other hand, the biochemistry and architecture of algal cell walls differ substantially from those of land plants. Briefly, as shown in Table 1, the major polysaccharide classes reported for many charophytes are cellulose, mannan (a hemicellulose) and homogalacturonan (HG; a pectic domain), but the results of conventional biochemical analysis and immuno‐labelling do not always match. It seems that typical land‐plant polysaccharides such as xyloglucan, xylans and rhamnogalacturonan‐I (RG‐I) are not equally distributed (or present in detectable quantities) in charophytic algae. This implies that there must be a broad diversity of poorly understood wall polymers in charophytes.

**TABLE 1:** Major differences between land‐plants and charophytes in their cell‐wall‐related polymers.

Polymer class	Polymer	Presence reported in		
		Land‐plants	Charophytes (by chemical/enzymic/molecular/histochemical evidence)	Charophytes by immuno‐based evidence (microarrays, microscopy)
Cellulose	Cellulose	**+**	**+**	**+**
Hemicelluloses	Xyloglucan	**+**	**±** *Mesotaenium*, *Netrium* ^4^; *Spirogyra* ^6^	**±** *Chara* ^16^; *Cylindrocystis*, *Netrium*, *Mesotaenium*, *Coleochaete* ^4^ ^,^ ^7^; *Zygnema* ^5^; *Spirogyra* ^14^
	1,3;1,4‐β‐Glucan	**±** *	**±** *Cosmarium*, *Chlorokybus*, *Klebsormidium* ^18^; *Mesotaenium* ^17^; *Chara* ^12^ ^,^ ^18^ ^,^ ^19^	**±** *Cosmarium*, *Microsterias*, *Pleurotaenium* ^7^; *Klebsormidium*, *Zygnema* ^5^
	Xylans	**+**	**±** *Nitella*, *Chara* ^8^ ^,^ ^12^ ^,^ ^18^ ^,^ ^19^; *Coleochaete* ^8^; *Klebsormidium* ^3^	**±** *Chara*, *Cosmarium* ^7^; *Klebsormidium* ^3^ ^,^ ^5^; *Spirogyra* ^14^
	Mannans	**+**	**±** ^1^ ^,^ ^2^ ^,^ ^8^ ^,^ ^12^ ^,^ ^16^ ^,^ ^18^ ^,^ ^19^; Not detected in *Coleochaete* ^8^	**±** ^1^ ^,^ ^2^ ^,^ ^4^ ^,^ ^7^ ^,^ ^14^ ^,^ ^16^; Not detected in *Chlorokybus* ^7^
Pectic domains	Homogalacturonan	**+**	**+** Except in *Klebsormidium* ^8^ & *Chlorokybus* ^20^	**+** Except in *Klebsormidium* & *Chlorokybus* ^7^
	RG‐I	**+**	**−**	**±** *Penium* ^15^
	RG‐II	**+**	**−**	**−**
	Galactans	**+**	**−**	**±** *Chara*, *Nitellopsis*, *Spirogyra* ^10^ ^,^ ^11^
	Arabinans	**+**	**−**	**±** *Chara* ^16^; *Netrium* ^7^ ^,^ ^10^ ^,^ ^11^; *Nitellopsis* ^10^ ^,^ ^11^
Non‐polysaccharides	Extensins	**+**	**±** *Klebsormidium* ^13^; *Spirogyra* ^14^	**±** *Netrium* ^7^; *Spirogyra* ^14^
	AGPs	**+**	**±** Not detected in *Nitella* & *Nitellopsis* ^10^ ^,^ ^11^, **	**±** ^1^ ^,^ ^2^ ^,^ ^7^ ^,^ ^14^ ^,^ ^16^; Not detected in *Nitella* & *Nitellopsis* ^10^ ^,^ ^11^, **
	1,3‐β‐Glucan	**+**	**±**	**±** *Coleochaete*, *Chlorokybus*, *Klebsormidium* ^7^; *Penium*, *Chara* ^16^
	Lignin	**+**	**±** *Zygogonium*, *Zygmena*, *Spirogyra* ^9^, ***	**–** Except *Coleochaete* ^7^, ***

**+**, generally present; **−**, generally absent; **±**, present only in certain taxa, e.g. those named. Adapted from [1] and [2]; supplemented and updated by the studies of [3]‐[20].

* Poales and Equisetales only; also present in lichens.

** Charophytes possess atypical AGPs with low Ara content or no hydroxyproline.

*** Phenolics only.

References:

^1^ Popper & Tuohy, 2010;

^2^ Domozych et al., 2012;

^3^ Jensen et al., 2018;

^4^ Mikkelsen et al., 2021;

^5^ Herburger et al., 2018;

^6^ Ikegaya et al., 2008;

^7^ Sørensen et al., 2011;

^8^ O'Rourke et al., 2015;

^9^ Permann et al., 2022;

^10^ Pfeifer et al., 2022;

^11^ Pfeifer et al., 2023;

^12^ Franková & Fry, 2021;

^13^ Liu et al., 2016;

^14^ Permann et al., 2021;

^15^ Palacio‐Lopez et al., 2020;

^16^ Domozych et al., 2010;

^17^ Eder et al., 2008;

^18^ Kiemle 2010;

^19^ Present study;

^20^ Rapin et al., 2023

### 1.1 Land‐plant polysaccharides

The three major polysaccharide classes in land plants are pectin, hemicelluloses and cellulose. It cannot be expected that the structures of these will be precisely conserved between charophytes and land‐plants; nevertheless, all three classes are present in *Chara* and *Arabidopsis*, and a brief description of these polysaccharide classes in land‐plants will set the scene for *Chara*.

Land‐plant pectin is a complex, multi‐domain polysaccharide that is, by definition, rich in α‐d‐galacturonic acid (GalA) residues. The three major domains are HG, RG‐I and RG‐II, which can readily be distinguished after digestion of whole cell walls with endo‐polygalacturonase (EPG). This enzyme cleaves the HG domains (after pre‐treatment with mild alkali to remove the methyl and acetyl ester groups) to yield GalA_1_, _2_ and _3_, and in the process also releases intact RG‐I (M_r_ ~ 50–500 kDa; Kaczmarska et al., 2022) and RG‐II (M_r_ ~ 5 kDa; Kobayashi et al., 1999; Begum & Fry, 2023). It is suspected that land‐plant pectin comprises a large polysaccharide in which HG, RG‐I and RG‐II are glycosidically linked like beads on a string. Thus, whole pectin molecules can be cross‐linked via any of their domains — for example, Ca^2+^ bridges (between HG domains) and borate bridges (between RG‐II domains; Sanhueza et al., 2022), and also potentially via xyloglucan–RG‐I glycosidic bonds (Thompson & Fry, 2000), RG‐I–cellulose bonds (Lin et al., 2016) and GalA–polyamine amide bonds (Perrone et al., 1998).

Of the hemicelluloses, mannans are predominantly polymers with a linear backbone of β‐1,4‐linked mannosyl residues (Moreira & Filho, 2008). The backbone may contain only mannose (Man; ‘pure mannan’) or both glucose (Glc) and Man residues (glucomannan) and occasional side chains of α‐1,6‐linked galactose (Gal) residues (galactomannan / galactoglucomannan; hetero‐mannans; Petkowicz et al., 2001; Voiniciuc, 2022). Both the Man and Glc residues can bear *O*‐acetyl groups (e.g. *O*‐acetyl‐galactoglucomannan). Mannans from algae and land plants differ from those of fungi and bacteria, which are α‐linked. Similar to pure mannans, glucomannans also provide structural support to the cell wall, for example, in hardwood (angiosperm) or softwood (gymnosperm) fibres (Fengel & Wegener, 1984). On the other hand, Galactomannans tend to be storage polysaccharides found in the endosperm of Fabaceae species and underground storage organs of some monocots (Bento et al., 2013; Matheson, 1990).

Xylans are the second most abundant plant polymers on Earth (Scheller and Ulvskov, 2010). The xylan backbone is formed by β‐1‐4‐linked xylose (Xyl) residues. The main classes of xylan are glucuronoxylans (decorated with glucuronate residues), arabinoxylans (arabinofuranosyl‐substituted) and glucuronoarabinoxylans (branched with α‐l‐arabinofuranose (Ara) and glucuronate (GlcA) side chains; Curry et al., 2023; Smith et al., 2017). Glucuronoarabinoxylans are typical of grasses, cereals and palms, and are thought to be the main cellulose‐tethering hemicelluloses in both primary and secondary cell walls. A common feature of grass xylans is further substitution of Ara with feruloyl, coumaroyl and β‐xylosyl residues, which make a significant contribution to the stability of the plant cell wall and its resistance to digestion by microbes and herbivores (Buanafina, 2009).

Xyloglucan is a typical land‐plant hemicellulose which comprises a linear backbone of (1→4)‐linked β‐Glc residues, many of which carry an α‐Xyl residue on position 6 (Fry et al., 2011). Typically, every fourth Glc residue is unsubstituted, resulting in structures based on XXXG [for nomenclature, see Fry et al. (1993)]. Some of the Xyl residues (especially the third from the non‐reducing end) carry an additional β‐Gal residue on position 2, and the β‐Gal itself often carries α‐Fuc on its 2‐position (forming XLXG and XLFG, respectively). The detailed structure and sequence of diverse side chains along the backbone of xyloglucan has been reviewed (Fry, 2011; Franková & Fry, 2012b).

Mixed‐linkage β‐glucan (MLG) is a hemicellulose with an unbranched backbone of β‐d‐Glc residues that are interconnected by a majority of (1→4)‐linkages and a minority of (1→3)‐linkages. It occurs in the Poales (e.g. grasses and cereals; Albersheim et al., 2010) and Equisetales (horsetails; Fry et al., 2008a; Sørensen et al., 2008).

### 1.2 Charophyte polysaccharides

Late‐diverging charophytes (i.e., the classes Charophyceae, Coleochaetophyceae and Zygnematophyceae) possess pectic HG domains (for further details, see Table 1). At least one charophyte, *Penium*, also possesses an RG‐I‐cross‐reacting epitope (Domozych et al., 2014).

The presence and fine structure of hemicelluloses varies across plant taxa (Popper, 2008; Sarkar et al., 2009; Sørensen et al., 2010). Their evolution defined terrestrialisation (colonisation of the land by aquatic organisms) and plant diversification. Some marine algae lacking cellulose contain mannans that form crystalline fibrils functioning as structural skeletons (Mackie and Preston, 1968). Unbranched linear β‐mannans can be assembled into para‐crystalline microfibrils (as seen in Rhodophyta, red algae; Popper et al., 2011) and were also reported from some Chlorophyta (non‐streptophyte green algae), including the giant unicellular alga *Acetabularia* (Dunn et al., 2007). Mannans can sometimes function in place of cellulose, e.g. in the chlorophyte *Codium* (Fernández et al., 2015); they are mostly fibrillar and 2‐*O*‐sulphated, maintaining amorphous cell‐wall regions. It is believed that mannan‐abundant cell walls are more extensible (Rodríguez‐Garcio et al., 2012) and possibly more accessible to enzymic remodelling, e.g. by mannan transglycanase activities as reported by Franková & Fry (2021), than conventional cellulose‐rich cell walls. In contrast to mannan:mannan transglycanase activities, cellulose:cellulose transglycanases acting on native (crystalline) cellulose do not occur in the plant kingdom (Shinohara et al., 2017; further discussed below). Practically all charophytes contain mannans in their cell‐wall matrix (Domozych & LoRicco, 2023; O'Rourke et al., 2015; Permann et al., 2021); few exceptions exist (e.g. *Chlorokybus*; Sørensen et al., 2011) (Table 1).

Charophycean cell walls contain unsubstituted xylans and ‘decorated’ arabinoxylans (detection mostly based on the xylan‐derived antibody approach; occasionally proven by biochemical analyses, O'Rourke et al., 2015; Popper & Fry, 2003). The exact type of xylan differs between charophytes. For example, *Chara aspera* (Charales) cell walls lack xylan, *C. corallina* and *Klebsormidium flaccidum* (Zygnemataleas) have only highly substituted, but no linear xylan, and other representatives of Charales (*C. globularis*, *C. suspinosa* and *C. tomentosa*) contain unsubstituted xylans only (Pfeifer et al., 2023).

The most controversial hemicelluloses In charophytes are xyloglucan and MLG. In contrast to land plants, charophycean algae contain xyloglucan‐like polymers that are chemically similar (composed of both Glc and Xyl residues) but structurally different (concerning frequency and linkage in side chains) from those of land plants. For example, a xyloglucan‐like polysaccharide was reported in *Spirogyra* (Ikegaya et al., 2008); and a xyloglucan‐like polysaccharide from *Coleochaete arbicularis* and *Cylindrocystis brebissonii* was thought to be composed of unusual repeat units e.g. LGGGG, PGGGG and LPGGG (Mikkelsen et al., 2021), where G stands for Glc, L for β‐Gal‐(1→2)‐α‐Xyl‐(1→6)‐substituted Glc and P for β‐GalA‐(1→2)‐β‐Gal‐(1→4)‐substituted Xyl attached to Glc via an α‐(1→6) bond. The “P”‐containing motif (in XLPG) was also proposed for *Mesotaenium caldariorum* (Mikkelsen et al., 2021) (Table 1).

### 1.3 Enzymic transglycosylation reactions

This paper focuses on wall‐localised enzymes that catalyse transglycosylation. Transglycosylation is a reaction, often enzyme‐catalysed, that cleaves a glycosidic bond (in a ‘donor substrate’) and conserves the energy of that bond in making a new glycosidic bond (to an ‘acceptor substrate’). Transglycosylation may be described as ‘cutting and pasting’ of sugars (Franková & Fry, 2013). In some cases, the energy of the cut bond greatly exceeds that of the new bond that is made, such that the reaction is essentially irreversible. Classic examples of this situation are in polysaccharide synthases, where the donor is a nucleoside diphosphate sugar (e.g. UDP‐glucose; thus, the linkage that is broken is a sugar–phosphate bond), and the product is a new sugar–sugar linkage (e.g. within a polysaccharide). In the present paper, however, we focus on cases where the energy of the broken bond approximately equals that of the newly produced bond, such that the reaction is freely reversible. Such reactions can be summarised as A–B + C ↔ A–C + B, where A, B and C are sugar residues: A–B is the donor substrate, C is the acceptor substrate, A–C is the ‘hybrid product’, and B is the leaving group. In principle, A, B and C can be identical. Such cutting and pasting reactions, while not necessarily causing any chemical change, can re‐structure cell wall polysaccharides and are thus of great interest in understanding changes that can occur in the plant cell wall, potentially controlling cell growth and wall assembly.

Cell‐wall polysaccharides that can serve as donor substrates in the ‘readily reversible’ type of transglycosylation reaction include xyloglucan, MLG, cellulose, mannan and xylan (Franková & Fry, 2013). Often, the acceptor substrate is similar or identical to the donor; enzymes catalysing such reactions are thus called homo‐transglycosylases, examples (expressed as ‘donor: acceptor’) being xyloglucan:xyloglucan (Fry et al., 1992), mannan:mannan (Schröder et al., 2006) and xylan:xylan (Franková & Fry, 2011; Johnston et al., 2013) homo‐transglycanases, the activities involved in these three cases being xyloglucan endotransglucosylase [XET, or trans(xylo)glucanase], trans‐β‐mannanase and trans‐β‐xylanase respectively. In these examples, the bond cleaved is a mid‐chain linkage in the polysaccharide chain, and the enzyme is thus a transglycanase (a.k.a. endo‐transglycosylase). [Note: ‘glyco’ implies an unspecified sugar residue, whereas ‘gluco’ specifically implies a Glc residue.] Concerning a hypothetical cellulose:cellulose homo‐transglucanase activity, we note that arabidopsis XTH3 exerts transglucanase activity with cellohexaose or artificially H_3_PO_4_‐amorphised cellulose (but not native, crystalline cellulose) as the donor substrate, and cello‐oligosaccharides (but not cellulose) as the acceptor substrate (Shinohara et al., 2017); it is thus difficult to propose that it acts on (crystalline) cellulose in plant cell walls.

In other cases of homo‐transglycosylases, the bond cleaved is a non‐reducing terminal linkage in the polysaccharide chain, and the enzyme is thus a transglycosidase (a.k.a. exotransglycosylase). Examples of the latter type of activity are trans‐α‐xylosidase (transferring a single α‐Xyl residue from one xyloglucan molecule to another; Franková & Fry, 2012a,b), trans‐β‐xylosidase (transferring a single β‐Xyl residue from one xylan molecule to another; Franková & Fry, 2021) and trans‐β‐galactosidase (transferring a single β‐Gal residue from one xyloglucan molecule to another; Franková & Fry, 2012a).

It may be envisaged that a transglycanase (endo‐activity) could, by a single catalytic event, exert a profound effect on the mechanics of the cell wall, greatly enhancing wall extensibility, for example. In contrast, a transglycosidase (exo‐activity) would have only a small direct mechanical effect, though potentially a major knock‐on effect by altering the ability of a whole polysaccharide molecule to serve as an acceptor substrate for a transglycanase (Franková & Fry, 2011).

Besides homo‐transglycosylases, plants also possess hetero‐transglycosylase activities. In particular, an enzyme exists (hetero‐trans‐β‐glucanase; HTG; Simmons et al., 2015) in *Equisetum* that can use MLG or cellulose as the donor substrate and xyloglucan as the acceptor, creating a ‘hybrid product’ that is MLG–xyloglucan or cellulose–xyloglucan. Such enzyme activities can be named, for example, MLG:xyloglucan endotransglucosylase (MXE or MLG: xyloglucan transglucanase; Fry et al., 2008b) and cellulose:xyloglucan endotransglucosylase [CXE, or cellulose:xyloglucan transglucanase, acting on native crystalline cellulose (Simmons et al., 2015 and on artificially H_3_PO_4_‐amorphised cellulose (Shinohara et al., 2017)]. In some cases, the hetero‐transglycosylase activity is the preferred activity (e.g. Simmons et al., 2015, where MXE activity exceeds XET activity), whereas in others it is a side‐reaction occurring at a low rate [e.g. MXE:XET activity ratio ~ 1:500, catalysed by a barley XTH (Hrmova et al., 2007), the reaction being undetectable in barley in vivo (Mohler et al., 2013)].

Heterotransglycanases can be of great interest in that they interlink dissimilar polysaccharides within the cell wall — for instance bonding cellulose to xyloglucan and thus potentially anchoring xyloglucan to the cellulosic microfibrils (Herburger et al., 2021).

Another variable among transglycosylases is their hydrolysis:transglycosylation ratio. Some transglycosylases can catalyse hydrolysis of the donor substrate (such a reaction can be thought of as ‘transglycosylation’ with H_2_O as the acceptor substrate) in addition to classic sugar–sugar transglycosylation, whereas others are confined almost exclusively to transglycosylation. Of the transglycanases (i.e., endo‐enzymes), most XET‐active enzymes are strict transglycosylases, but a small proportion of them (e.g. AtXTH31; Shi et al., 2015) are principally hydrolases. Trans‐β‐mannanases (Schröder et al., 2006) and trans‐β‐xylanases (Franková & Fry, 2011; Derba‐Maceluch et al., 2015) appear to catalyse principally “non‐mechanistic” glycosyl transfer as their products remain stable without being reversibly hydrolysed and are referred to as “dedicated transglycanases” (Franková & Fry, 2013). Many transglycosidases (i.e., exo‐enzymes) catalyse both transglycosylation and hydrolysis, with the former reaction type being favoured at high acceptor substrate concentrations, although some trans‐α‐xylosidases exhibit a remarkably high transglycosylation:hydrolysis ratio even at low substrate concentrations (Franková & Fry, 2012a).

### 1.4 Transglycosylation reactions in charophyte cell walls

The great majority of information on wall‐localised transglycosylases (catalysing reversible or dedicated transglycosylation reactions) comes from land‐plants, especially angiosperms, with few studies having focused on algae (Fry et al., 2008b; van Sandt et al., 2007; Herburger et al., 2018; Franková & Fry, 2021). In the present paper, we explore to what extent comparable activities may occur in a charophytic cousin of the land plants — *Chara vulgaris*, a representative of the Charophyceae. The aim is to explore the ancient evolutionary origin of wall‐remodelling activities among the streptophytes.

In this study, we focus on profiling and characterising transglycanase and transglycosidase activities that modify mannans and xylans – two main hemicelluloses abundant in charophycean algae. We aim to find out whether both TBM (trans‐β‐mannanase) and TBX (trans‐β‐xylanase) can ‘cut and paste’ mannans, xylans and other polymers, thus potentially affecting charophyte wall assembly, strength and mechanical properties.

## 2 MATERIALS AND METHODS

### 2.1 Polysaccharide and oligosaccharide substrates

1,4‐β‐Mannan (borohydride reduced, non‐acetylated; *Ceratonia siliqua*, catalogue reference P‐MANCB, degree of polymerisation (DP) ~15, M_r_ ~ 2500), arabinoxylan (*Triticum aestivum*, P‐WAXYM, medium viscosity, M_r_ ~ 300,000, Ara:Xyl 38:62, DP ~1500), lichenan (1,4:1,3‐β‐linkage ratio ≈ 2:1; *Cetraria islandica*, P‐LICH, DP ~1700, M_r_ ~ 275,000), glucomannan (low viscosity, partially acetylated; *Amorphophallus konjac*, P‐GLCML, M_r_ 950,000, DP ~5000–5500, Man:Glc = 6:4), and mixed‐linkage glucan (1,4:1,3‐β‐linkage ratio ≈ 3:1; medium viscosity, *Hordeum vulgare*, P‐BGBM, DP ~1500, M_r_ ~ 245,000), linear arabinan (*Beta vulgaris*, P‐LARB, Mr ~18,000, 97.5% Ara), arabinan (*Beta vulgaris*, P‐ARAB, 88% Ara), galactan (*Solanum tuberosum*, P‐GALPOT), galactomannan (Guar, medium viscosity, P‐GGMMV, Gal:Man = 38:62, Mr ~380,000), rhamnogalacturonan‐I (RG‐I; *Solanum tuberosum*, P‐RHAM1), galactomannan (*Ceratonia siliqua*, low viscosity, P‐GALML, M_r_ ~ 107,000, Gal:Man = 24:76), mannohexaose (Man_6_) and xylohexaose (Xyl_6_) were from Megazyme (http:// www.megazyme.com). Xylan (birchwood, X‐0502, Xyl≥90%; M_r_ ~ 98,000), 4‐*O*‐methyl‐d‐glucuronoxylan (*Fagus sylvatica*, M‐5144; 96% soluble, 13% hexuronic acids, the rest being mainly Xyl; Hespell and Cotta, 1995), fucoidan (*Fucus vesiculosus*, F‐5631), λ‐carrageenan (*Gigartina aciculaire* and *G. pistillata*, C‐3889, type IV, non‐gelling), arabinogalactan (*Larix occidentalis*, A2012), pectin (esterified, *Citrus* sp., P9561) and homogalacturonan (polygalacturonic acid – PGA, P7276) was obtained from Sigma. Sodium alginate (*Laminaria hyperborea*, 30105) was from BDH Chemicals. Tamarind xyloglucan (*Tamarindus indica*, backbone DP ~ 3000, M_r_ ≈ 1,000,000) was donated by Dainippon Pharmaceutical Co., Osaka. Water‐soluble cellulose‐acetate was prepared in‐house, according to Fry et al. (2008a). Man_6_‐ol–SR [the sulphorhodamine conjugate of Man_6_, prepared as described by Miller et al. (2007)], [^3^H]XXXGol [borohydride‐reduced xyloglucan heptasaccharide (Xyl_3_.Glc_3_.glucitol)], [^3^H]Man_6_‐ol (mannohexaitol) and [^3^H]Xyl_6_‐ol (xylohexaitol) (each with specific activity ~900 MBq/μmol) were obtained from EDIPOS (http://fry.bio.ed.ac.uk/edipos.html). [The system of nomenclature for xyloglucan oligosaccharides (XXXGol etc.) is explained by Fry et al. (1993).]

### 2.2 Plant material

An axenic culture of *Chara vulgaris* was kindly provided by Prof. Burkhard Becker (Cologne University) and cultured unshaken in BBS (Bold's Basal medium plus Soil extract; http://cshprotocols.cshlp.org/content/2018/11/pdb.rec102871.full?text_only=true) at 23.5°C under continuous moderate illumination (photosynthetic photon flux density = 500 μmol m^−2^ s^−1^), provided by Philips TLD 36 W/830 fluorescent tube, Warm White 3000 K).

### 2.3 Enzyme preparation

Enzyme extracts of axenic *Chara* were prepared as described by Franková & Fry (2021); all steps were performed at 0–4°C. Algal cultures were thoroughly rinsed with de‐ionised water and then vigorously homogenised by mortar and pestle in extraction buffer (0.3 M succinate [Na^+^, pH 5.5] supplemented with 15% v/v glycerol and 20 mM ascorbate) containing 3% (w/v, suspension) polyvinyl polypyrrolidone at an extraction ratio of 1:4 (g fresh weight:ml extractant). After 3 h of gentle stirring, the homogenate was filtered through three layers of nylon gauze and then centrifuged at 10,500 *g* for 45 min. For the isoelectric‐focusing experiment, a different extract was prepared: axenic algal cultures were freeze‐dried and then extracted with 0.2 M succinate (Na^+^, pH 5.5) containing 1 M NaCl, 0.02% Triton and 5% glycerol. The extraction ratio varied from 1:10 to 1:20 (g freeze‐dried culture: ml extractant) according to the density of the final homogenate. After homogenisation with a pestle and mortar followed by 3 h stirring, the homogenate was centrifuged (10,500 *g*, 45 min) and dialysed twice against 2.5 mM succinate [Na^+^, pH 5.5].

### 2.4 Isoelectric focusing

Extract containing 5% glycerol and 2% ampholites (Bio‐Lite 3–10, 163–1112, BioRad Inc.) was isoelectric‐focused in a Rotofor Cell IEF (isoelectric focusing) apparatus (BioRad) according to the manufacturer's instructions. Electrophoresis was performed at 12 W until the voltage and current stabilized. The pH of the fractions was then measured, the fractions were de‐salted on a centrifugal concentrator (Vivaspin, 5 kDa molecular weight cut off) and 5 μL was assayed for transglycanase activities.

### 2.5 Transglycanase (endo‐) activity assays

The following transglycanase substrates were employed for assaying transglycanase activities: tamarind xyloglucan, nasturtium xyloglucan, MLG, lichenan, arabinoxylan, 1,4‐β‐xylan (birch wood), xylohexaose, mannohexaose, SR‐labelled xylan, glucuronoxylan, arabinan, linear arabinan, glucomannan, galactomannan, galactan, rhamnogalacturonan‐I (RG I), esterified pectin, water‐soluble cellulose‐acetate, homogalacturonan (‘polygalacturonic acid (PGA)’), fucoidan, carrageenan, alginate, and 1,4‐β‐mannan (all as donors), and [^3^H]XXXGol, [^3^H]Man_6_‐ol, and [^3^H]Xyl_6_‐ol (as acceptors). All polysaccharides were pre‐washed in 70% ethanol and then solubilised by vigorous stirring and boiling for 10 min except for 1,4‐β‐mannan, which was solubilised in 2.5 M NaOH and then neutralised with 50% acetic acid immediately before use. Commercial Man_6_ (mannohexaose; Megazyme) was freed of contaminating oligosaccharides by gel‐permeation chromatography (on Bio‐Gel P‐2 and P‐10). Oligomannans were eluted in pyridine/acetic acid/0.5% chlorobutanol in water (1:1:98) and individual fractions were profiled by thin‐layer chromatography (TLC; Frankova & Fry, 2011).

The standard reaction mixture contained 2 kBq (≈ 2 pmol) tritiated oligosaccharide (acceptor; dried in a PCR tube), 5 μL 1.2% (w/v) polysaccharide in 0.2 M succinate buffer (Na^+^, pH 5.5) and 15 μL enzyme extract in a final volume of 20 μL. Final concentrations were thus routinely 0.1 μM oligosaccharide, 0.3% w/v polysaccharide and 50 mM succinate. After 24 h incubation at 22°C, the reaction was stopped by addition of 4 μL formic acid. A control sample contained all components with either heat‐ or formic acid‐inactivated enzyme extract.

For testing optimal substrate concentrations, the reaction mixtures (final volume 10 μL) contained the following combination of substrates:

0–0.5% glucomannan + 0.2 μM [^3^H]Man_6_‐ol (used in Figure 4A)
0–0.5% 1,4‐β‐mannan + 0.2 μM [^3^H]Man_6_‐ol (used in Figure 4B)
0–0.3% (0–3.0 mM) Man_6_ + 0.1 μM [^3^H]Man_6_‐ol (used in Figure 4C)
0–0.4% 1,4‐β‐xylan + 0.1 μM [^3^H]Xyl_6_‐ol (used in Figure 4D)
0.3% glucomannan + 0.005–0.6 μM [^3^H]Man_6_‐ol (used in Figure 5A)
0.3% 1,4‐β‐xylan + 0.005–0.6 μM [^3^H]Xyl_6_‐ol (used in Figure 5B)

After incubation for 2 h, the reactions were stopped by addition of 6 μL of 15 M ammonia, which does not affect mannan solubility. The reactions for time‐courses (used in Figure 8) were stopped after 0–32 h.

The ^3^H‐labelled transglycosylase products were separated from unreacted oligosaccharides by a glass‐fibre blotting method (Franková & Fry, 2020) except for the products generated from Man_6_ or Man_6_‐ol, which were separated by the TLC method followed by ^3^H‐scanning (Franková & Fry, 2011; used in Figure 4C, Figure 6). The error bars represent three to four technical replicates (n = 3–4) ± SE.

### 2.6 Transglycosidase (exo‐) activity assays

The reaction mixture contained 7.5 μL enzyme extract, 1 kBq tritiated acceptor plus potential oligosaccharide donor substrate (final concentration 0.3%, or 0–0.3% in the case of assaying kinetic properties of TBM); final volume 10 μL. After 24 h incubation at 22°C, the reaction was stopped with 3 μL formic acid. The whole reaction mixture was dried on to a silica‐gel TLC plate and developed in butan‐1‐ol/acetic acid/water (BAW, 2:1:1 by vol.), with three ascents, except for Figure 9 where one ascent was employed. The dried TLC plate was fluorographed (Fry, 2000) and quantitatively profiled by 60‐min counting in an AR2000 radioisotope scanner (LabLogic; http://www.lablogic.com), revealing ^3^H‐labelled products. The same TLC plate was stained with thymol, revealing total sugar moieties (Franková & Fry, 2021).

### 2.7 Microscopy of *Chara* specimens and *in situ* detection of transglycanase activities

Non‐fixed, unlabelled *Chara* specimens were photographed with a Leica camera using Leica LAS AF software. *In‐situ* detection of transglycanase action on native algal walls was performed by use of fluorescently labelled acceptor substrate (Man_6_‐ol–SR). Water‐washed algal specimens were incubated in fresh algal culture medium containing 5 μM fluorescently labelled acceptor substrates for 6 h, then rinsed with ethanol/formic acid/water (5:0.5:4.5; 2 × for 20 min). This procedure removes all unreacted fluorescent acceptor substrates. The specimens were then rinsed with 5% formic acid (gentle rotation overnight) then briefly rinsed in water and mounted onto microscopy slides containing a drop of 5% glycerol. The fluorescence was visualised with a fluorescence microscope (Leica DM2000 LED) using a standard setting for all specimens. Controls contained boiled *Chara* samples (105°C for 1 h), which were incubated in fluorescent substrates and subjected to the same procedure as above.

### 2.8 Dot‐blot assay of TBM

Enzyme extract (5 μL) was loaded onto dry dot‐blot papers obtained from EDIPOS. [Briefly, the dot‐blot paper (marked with a grid in 96‐well plate format) was prepared by dipping Whatman No. 1 paper through 0.2% KGM (depositing ~36.6 μg per 9 × 9 mm square of paper) in 0.2 M succinate (Na^+^) buffer pH 5.5 containing 10 mM CaCl_2_ followed by dipping in 2.4 μM Man_6_–SR dissolved in 50% acetone (twice, final amount deposited was 0.094 nmol / 9 × 9 mm).] After 24 h incubation under humid conditions, the dot‐blot was dried, washed with 80, 60, 40, 20 and 10% EtOH supplemented with 0.5% acetic acid, each for 1 h, and documented in a Doc‐It imaging system (CamLab) under a 254‐nm UV lamp.

## 3 RESULTS

### 3.1 Choosing *Chara* for enzyme screening

**FIGURE 1:**
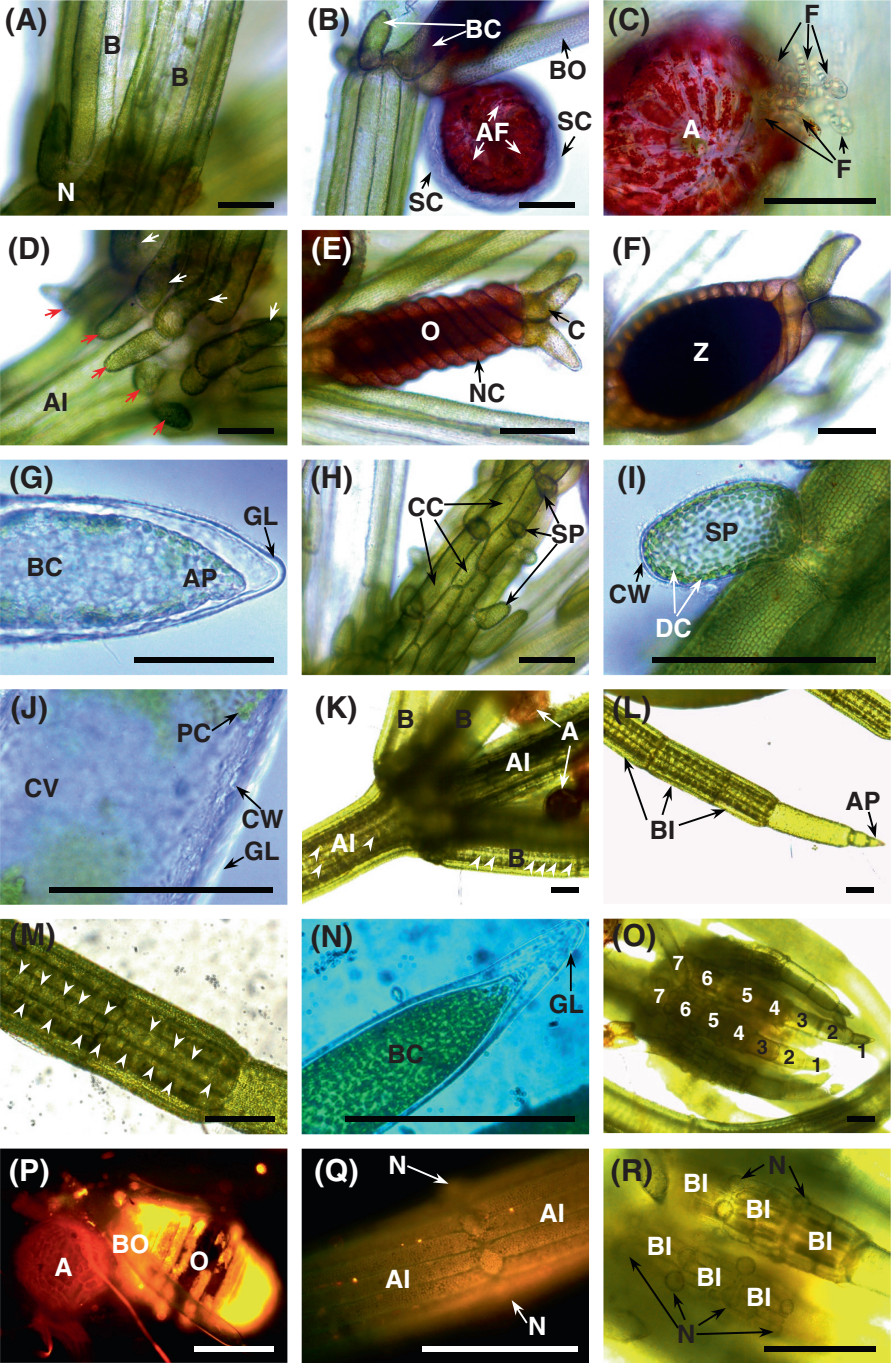
Microscopy of *Chara vulgaris* showing characteristic features of wild *Chara vulgaris* and laboratory‐maintained cultures. Specimens (A–J) grown under laboratory axenic and (K–R) natural conditions. Light microscopy (A–O and R) and fluorescence microscopy (P and Q). (A) *Chara* branchlets (B; leaf‐like structures) growing from the node (N) of the first whorl; (B) antheridium with red antheridial filaments (AF) and transparent shield cells (SC); BO, bracteole; BC, bract cell; (C) detail of spermatogenous filaments (F) leaving the mature antheridium (A); (D) upper and lower row of stipulodes (white and red arrows respectively) at the multicellular node; AI, axial internode of giant cells (stem‐like structure); (E) oogonium (O) with nocule (NC) and corona (C); (F) zygote (Z); (G) apex (AP) of a bract cell (BC) with a gelatinous layer (GL); (H) axis cortex with spine cells (SP) and cortex cells (CC); (I) detail of a spine cell (SP) with thin cell wall (CW) and discoid chloroplasts (DC) located at the periphery of the cytoplasm; (J) detail of the bract cell showing central vacuole (CV), peripheral chloroplasts (PC), and the cell wall (CW) surrounded by a gelatinous mucilage layer (GL); (K) a branchlet whorl with antheridia (A); both branchlets (B) and main axial internodes (AI) are covered by crystals of calcium carbonate; (L) lateral branchlet apex (AP; free of calcium crystals) and calcified branchlet internode cells (BI); (M) a detail of a branchlet internode with crystals of calcium carbonate (white arrows); (N) bract cell (BC) with a ‘talon’‐like ending coated with a mucilage layer (GL); (O) multicellular meristematic apex of ‘talon’‐like branchlets with corticate (4–7) and ecorticate (1–3) segments; (P) autofluorescence of antheridium (A) and oogonium (O); BO, bractiole; (Q) fluorescence micrographs of axial internode cells (AI) with a nodal zone (N); (R) a detail of branchlet apex with short branchlet internodes (BI) and numerous meristematic nodal zones (N). Scale bar = 200 μm.

We selected healthy *Chara vulgaris* specimens grown under laboratory axenic and natural conditions. Light microscopy (Figure 1) showed that the selected specimens of axenic *Chara* exhibited typical features of wild‐grown specimens such as a well‐developed whorl of branchlets and stipulodes, cortex cells, crystals of calcium carbonate, characteristic red antheridia and oogonia, and numerous meristematic nodal zones. This means that our axenic cultures of *Chara vulgaris* were healthy and continued their life cycle as if grown in their natural freshwater habitat.

**FIGURE 2:**
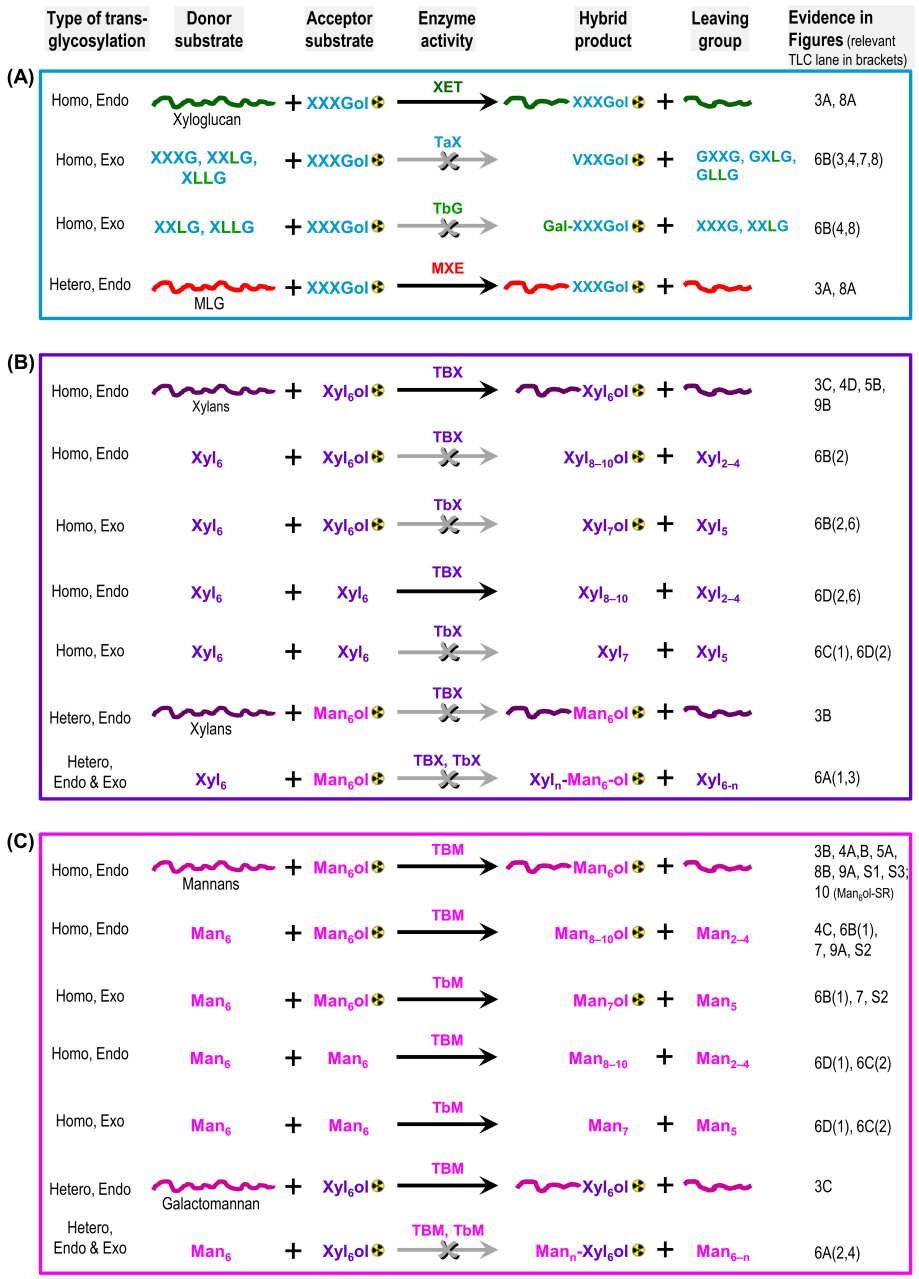
Schematic summary of *Chara* transglycanase and transglycosidase action on diverse xyloglucan‐, xylan‐ and mannan‐based substrates. (A) Donor‐to‐[^3^H]oligoxyloglucan transglycosylase activities; XET, xyloglucan endotransglucosylase, MXE, MLG:xyloglucan endotransglucosylase (hetero‐transglycosylase); TaX, trans‐α‐xylosidase; TbG, trans‐β‐galactosidase; endo, endo‐acting transglycanase, exo, exo‐acting; (B) xylan‐to‐[^3^H]oligoxylan and xylan‐to‐oligomannan transglycosylase activities; TBX, trans‐β‐xylanase; TbX, trans‐β‐xylosidase; hetero, hetero‐transglycosylase (xylan:mannan transglycosylase); (C) mannan‐to‐[^3^H]oligomannan and mannan‐to‐oligoxylantransglycosylase activities; TBM, trans‐β‐mannanase; TbM, trans‐β‐mannosidase.

In our earlier screens of numerous algal and fern extracts (Franková & Fry, 2021), we found that the Charophyceae, among other classes, contain extractable glycanase activities that are capable of *in‐vitro* remodelling of their endogenous cell‐wall polysaccharides as well as commercial extrinsic land‐plant‐derived mannan, xylan and (surprisingly) xyloglucan polysaccharides. As transglycanase and transglycosidase activities from *Chara* and *Nitella* were often higher than those in land plants, we decided to explore transglycanases in *Chara* in greater detail and to assess the potential of cell‐wall remodelling in charophytes and compare it with XET‐based xyloglucan remodelling in early‐ and late‐diverging land‐plants. Therefore, we prepared crude extracts from *Chara* using a ‘universal’ extractant (see material and methods) and screened a wide range of extrinsic land plant and algal polysaccharides, which could act as donor substrates for transglycanase activities in *Chara*. To help the reader navigate this paper, Figure 2 summarises the main activities tested for, including those that were and those that were not detected.

### 3.2 XET‐like and MXE‐like activities detected in *Chara* extracts

**FIGURE 3:**
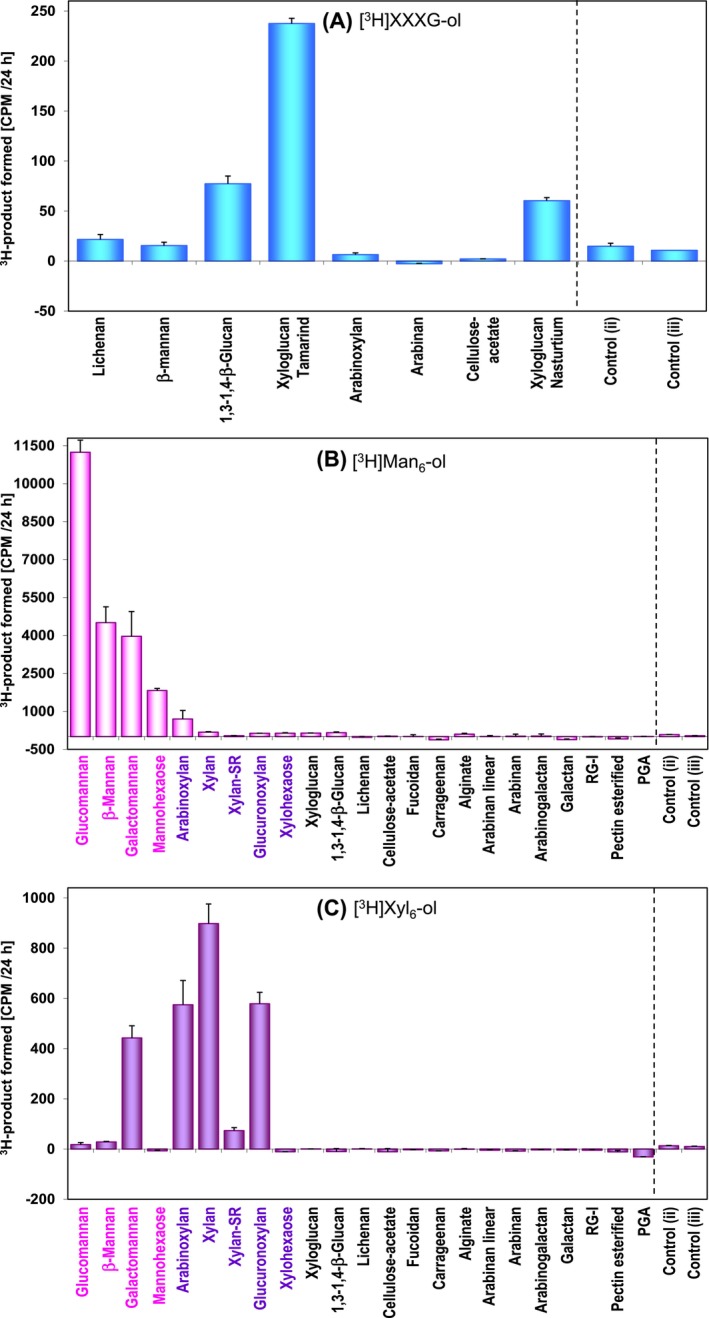
Profiling substrate specificities of extracted *Chara* transglycanases. Transglycanase activities of a *Chara* extract on diverse polysaccharides as potential donor substrates. The potential acceptor substrates tested were (A), [^3^H]XXXGol; (B), [^3^H]Man_6_‐ol; (C), [^3^H]Xyl_6_‐ol. Each reaction mixture contained 1–2 kBq of acceptor, 0.3% w/v donor, and *Chara* enzyme extract in a final volume of 10 μL. Enzymes had been extracted in 0.2 M succinate (Na^+^) buffer pH 5.5 in 1:4 ratio (w/v) or in 0.3 M succinate (Na^+^) buffer pH 5.5 enriched with 20 mM ascorbic acid and 15% glycerol. The transglycanase products were separated from unreacted products by the glass‐fibre blotting method except for those from water‐soluble cellulose acetate (A). Three controls were prepared – (i) with formic acid‐inactivated enzyme, (ii) donor free (to correct for endogenous substrate which might be possibly co‐extracted with proteins), and (iii) an enzyme‐free control (to correct for non‐enzymic trapping of acceptor in the donor). All values were corrected to control (i). Thus each reaction mixture had its own control for each donor). Both controls (ii) and (iii) are corrected to background and are thus higher than baseline; the value in control (iii) was subtracted from the value obtained with enzyme. Since control (ii) was minimal in A, B and C we conclude that our extracts had negligible co‐extracted polysaccharides with donor capability, and thus values shown in the histograms are reliable. Negative values in some samples are given by the slight hydrolytic activity of *Chara* extract hydrolysing the acceptor. So if control (i) was higher than the undenatured enzyme sample it was attributable to the trapping of ^3^H in the polysaccharide during the glass‐fibre blotting step. Error bars represent four technical replicates (n = 4) ± SE.

It is still not certain whether *Chara* cell walls contain any typical xyloglucan or MLG (well‐known land‐plant polysaccharides, the former ubiquitous and the latter found only in *Equisetum* and the Poales; Table 1). Nevertheless, *in vitro*, endo‐transglycosylation could be found between xyloglucan and MLG (as donors) and tritiated oligoxyloglucan (as acceptor). If the transglycosylation occurs between xyloglucan and oligoxyloglucan, or between MLG and oligoxyloglucan, it indicates the presence of enzyme activities known as XET and MXE, respectively. Since the presence of conventional xyloglucan and MLG with typical linkages and structure as in land plants was not chemically proven in *Chara*, we will specify these activities as “XET‐ and MXE‐like” (Figure 3A). [It should be noted that we quote all the transglycosylation reactions as being ‘from’ donor ‘to’ acceptor.]

All the values were corrected to a control containing heat‐ or formic acid‐inactivated enzyme. The *Chara* XET‐like activity preferred tamarind xyloglucan over nasturtium xyloglucan (both non‐fucosylated, but whose major octasaccharides are XXLG and XLXG, respectively). MXE represented ~1/3rd of XET and was detectable on barley MLG (the donor substrate which comprises both cellotetraosyl and cellotriosyl β‐(1–3)‐linked repeat units) but only negligibly on *Cetraria* MLG (lichenan, which has almost only cellotriosyl repeats). No CXE (discovered in *Equisetum*; Herburger et al., 2020a; Simmons et al., 2015) was detected on an acetylated form of cellulose — water‐soluble cellulose acetate (Figure 3A).

### 3.3 Survey of extractable homo‐ and hetero‐transglycanase activities acting on hemicellulosic donor substrates

In *in‐vitro* assays, *Chara* extracts were tested for transglycanase activities with diverse polysaccharides as potential donors and either [^3^H]Man_6_‐ol or [^3^H]Xyl_6_‐ol as acceptors (Figure 3B,C).

With [^3^H]Man_6_‐ol as acceptor, the extracts exhibited TBM (homo‐trans‐β‐mannanase activity, i.e. with mannans as donor; Figure 3B). The activity was detected both with linear mannans (including Man_6_) and with two heteromannans, optimally with glucomannan.

With [^3^H]Xyl_6_‐ol as acceptor, the extracts exhibited TBX (homo‐trans‐β‐xylanase activity) with both unsubstituted xylan and heteroxylans (Figure 3C). TBX activity preferred pure unsubstituted xylan, but also acted on arabinoxylan ≥ glucuronoxylan and > > fluorescent (sulphorhodamine‐labelled) xylan. A short oligomannan (Man_6_) was a poor substrate for TBX owing the methodology used [glass‐fibre blotting method, detecting mostly polymeric ^3^H‐labelled products (Franková & Fry, 2015, 2020)]. Interestingly, substantial hetero‐trans‐mannanase activity (MXT) was detected with galactomannan as donor [(galacto)mannan:xylan transglycanase]. Negligible activity was found with other polysaccharides as potential donor (Figure 3C).

### 3.4 Non‐mechanistic trans‐β‐mannanase and trans‐β‐xylanase activities start at physiologically relevant donor substrate concentrations

**FIGURE 4:**
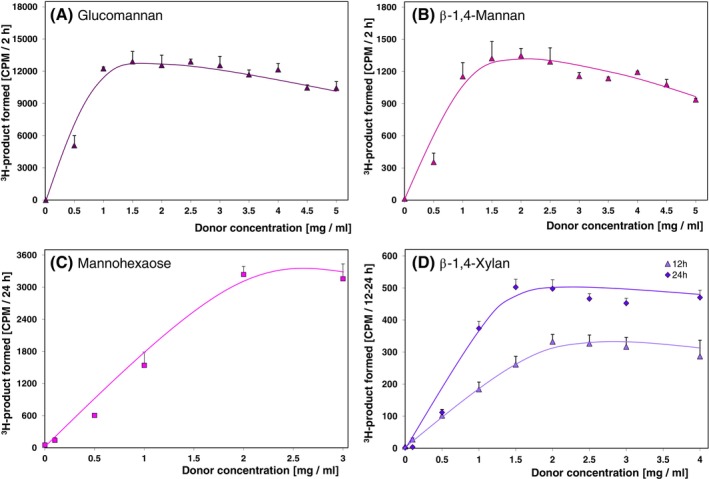
Dependence of the reaction rates on donor substrate concentrations. Trans‐β‐mannanase with donor substrates **(A)** glucomannan, **(B)** β‐1,4‐mannan, **(C)** mannohexaose; **(D)** trans‐β‐xylanase with donor substrate birchwood β‐1,4‐xylan The acceptor substrate was constant 100–200 nM [^3^H]Man_6_‐ol (**A–C**) or 100 nM [^3^H]Xyl_6_‐ol (**D**). All the activities were assayed by glass‐fibre blotting method except for those on mannohexaose where TLC approach followed by quantitative ^3^H‐scanning was employed. Error bars represent three technical replicates (n = 3) ± SE.

With their respective oligosaccharide acceptor substrates set at ~100 nM, both TBM and TBX were able to operate at the low donor substrate concentration of ~0.5 mg/mL (Figure 4). TBM also catalysed transglycosylation with non‐radioactive Man_6_ as donor (Figure 4C).

**FIGURE 5:**
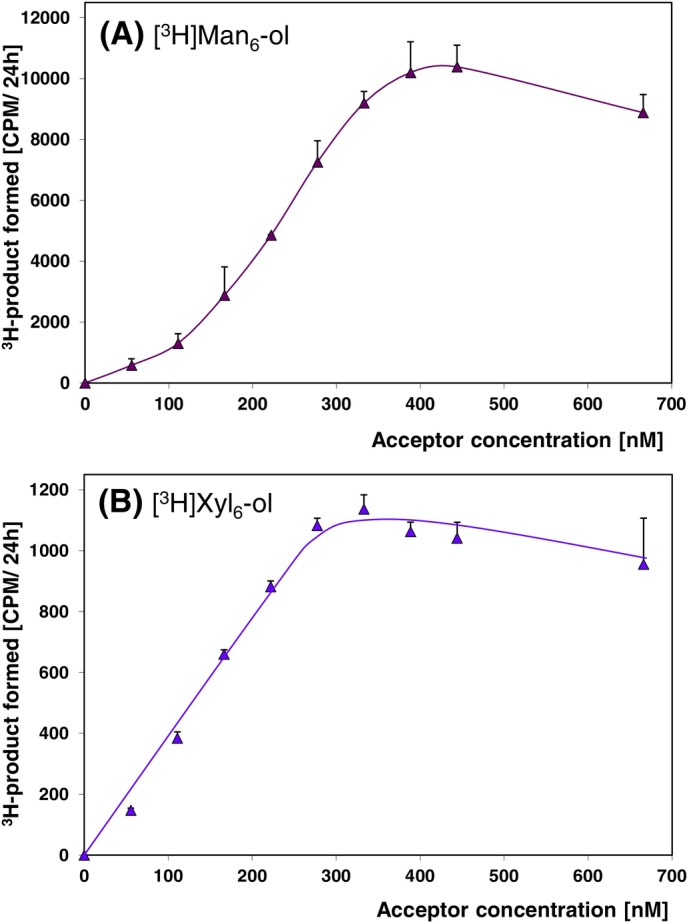
Dependence of the reaction rates on acceptor concentrations. (A) Trans‐β‐mannanase with constant 3 mg/mL glucomannan as donor and carrier‐free [^3^H]Man_6_‐ol as acceptor; (B) trans‐β‐xylanase with constant 3 mg/mL β‐1,4‐xylan as donor and carrier‐free [^3^H]Xyl_6_‐ol as acceptor. The incubation time for each assay was 24 h. Mean values ±SE (n = 3).

For both enzyme activities (TBM and TBX), the yield of product increased with acceptor substrate concentration, as expected (Figure 5). Surprisingly, the yield plateaued above ~300–400 nM concentration of acceptor substrate. This suggests that the *K*_M_ of these enzymes for their acceptor substrates is sub‐μM. The diminished activities seen at 700 nM acceptor concentration (Figure 5A,B) are likely to be caused by detection limit and sensitivity of scintillation counter used in detection of ^3^H‐labelled polymers.

*Chara* crude extract possessed high trans‐β‐mannanase activity but only barely detectable hydrolytic activity, unlike *Nitella*, which distinguishes *Chara* from its close relative *Nitella* (Figure S1).

**FIGURE 6:**
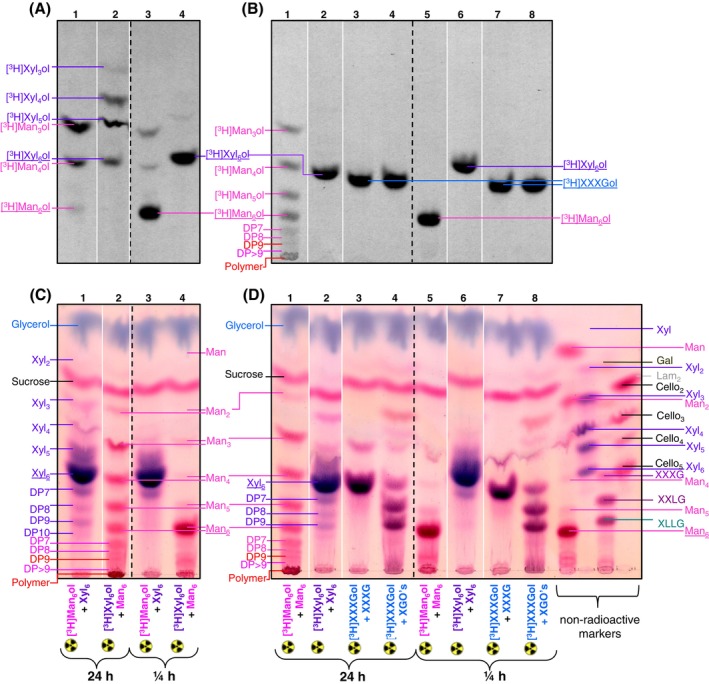
Testing hetero‐ and homo‐transglycosylase activities on model oligomeric substrates.Different reaction mixtures containing radioactive and non‐radioactive oligomers of mannan, xylan and xyloglucan were incubated with an enzyme preparation for 0.25 or 24 h, then analysed by TLC. (A,B) Fluorographic visualisation of ^3^H‐labelled oligosaccharides; (C,D) thymol‐stained non‐radioactive sugars on the same TLCs (compounds that are only radiolabelled are not visible in C and D). (A,C) Hetero‐transglycosylase activities; (B,D) homo‐transglycosylase activities. One of the two replicates of each TLC is shown.

**FIGURE 7:**
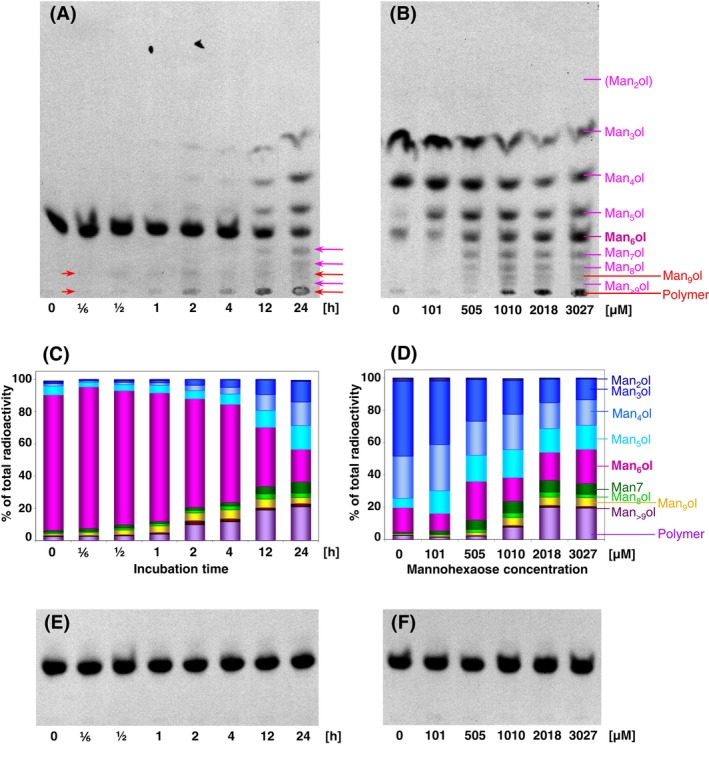
Kinetic properties of oligomannan‐remodelling transglycosylases extracted from *Chara vulgaris*. **(A)** Time‐dependent action of *Chara* transglycosylases and hydrolases on 0.1 μM [^3^H]Man_6_‐ol (acceptor) mixed with 3 μM Man_6_ (donor). Trans‐β‐mannanase products formed in the first 4 h are indicated with red arrows; trans‐β‐mannosidase products formed later have pink arrows. **(B)** Effect of substrate concentration on products formed by *Chara* transglycosylases and hydrolases after 24 h. Dried [^3^H]Man_6_‐ol was re‐dissolved at various concentrations (0–3027 μM) of Man_6_, mixed with *Chara* extract and incubated at 22°C for 24 h. **(C), (D)** The distribution of radioactivity between differently sized products on the TLCs shown in (A) and (B). The histogram bars represent the average of two replicates. The values were corrected to heat‐inactivated controls shown in Figure S4. **(E)** Controls with heat‐inactivated enzyme for (A); **(F)** controls with heat‐inactivated enzyme for (B).

The transglycosylation products of both TBM and TBX were relatively stable over time (up to at least 24 h; Figure 6), so it can be concluded that TBM and TBX catalyse non‐mechanistic transglycosylation that may have physiological relevance *in vivo*. The attachment of mannans to [^3^H]Man_6_‐ol was remarkable, especially that of glucomannan, where ~55% of initial acceptor provided became incorporated into polymers. The [^3^H]products formed with non‐radioactive Man_6_ were of higher molecular weight (~DP ≥10; Figures 7A,C; S2) thus detectable and were not hydrolysed. This suggests that the enzymes conserved in *Chara* are not just mannan‐ or xylan‐hydrolases with side, ‘mechanistic’ transglycanase activity but functional transglycosylases capable of remodelling mannan‐ and xylan‐rich cell walls.

### 3.5 The isoforms of *Chara* transglycanases

**FIGURE 8:**
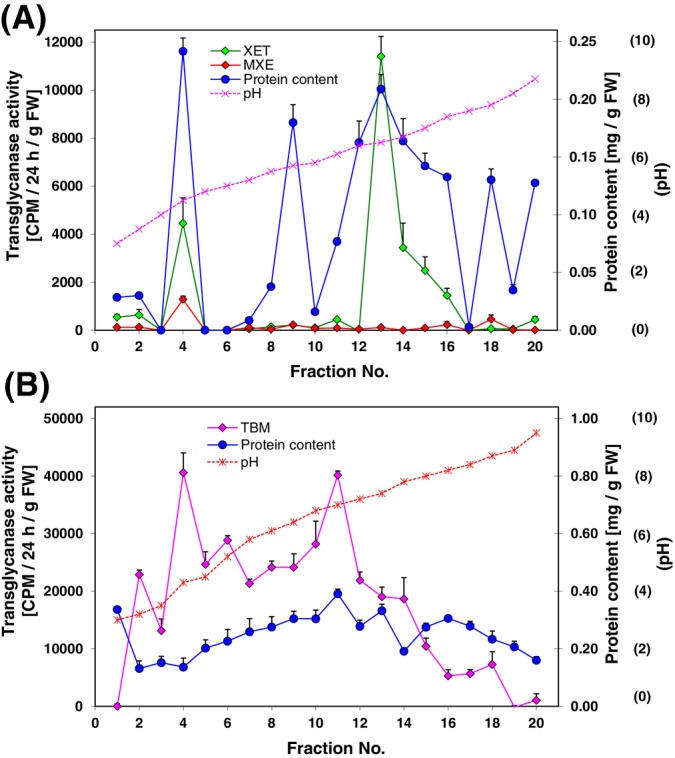
Profiling isoforms of transglycanases from *Chara* by isoelectric focusing.IEF fractions were assayed for (A) XET‐ and MXE‐like activities with 3 mg/mL xyloglucan or MLG as donor substrate and 0.2 μM [^3^H]XXXGol as acceptor and (b) TBM activity with 3 mg/mL β‐1,4‐mannan as donor and 0.2 μM [^3^H]Man_6_‐ol as acceptor. In all cases the reaction time was 24 h.

*Chara* transglycanases were also examined by isoelectric focusing (IEF, Figure 8). IEF revealed that *Chara* contains two isoforms of transglycanase acting on xyloglucan – one possessing XET‐like activity [the neutral isoform, pI (isoelectric point) = 7.4, in fraction 13 of Figure 8A] and one exhibiting both XET‐ and MXE‐like (the acidic isoform, pI = 4.3, in fraction 4) and thus capable of remodelling both xyloglucan and MLG. Likewise, TBM activity from *Chara* was present in at least two isoforms – neutral and acidic (pI = 7.0 and 4.3, in fractions 11 and 4, respectively, Figure 8B).

### 3.6 Transglycosidase (exo‐acting) activities on oligosaccharides

**FIGURE 9:**
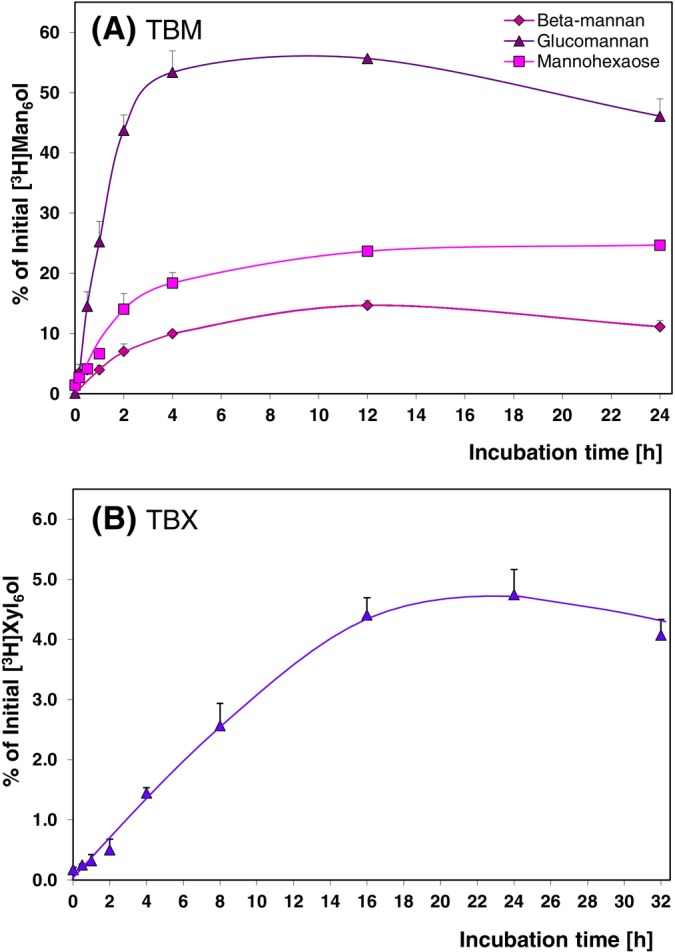
Time‐courses for *Chara* trans‐β‐mannanase and trans‐β‐xylanase activity *in vitro*.(A) Trans‐β‐mannanase with 3 mg/mL glucomannan, β‐1,4‐mannan or mannohexaose as donor substrate and 100 nM [^3^H]Man_6_‐ol as acceptor; (B) trans‐β‐xylanase with 3 mg/mL β‐1,4‐xylan as donor and 100 nM [^3^H]Xyl_6_‐ol as acceptor. Mean values ±SE (n = 3).

Using specific oligosaccharides to assay transglycosylase activities has the advantage over using polysaccharides in that the structures of the substrates are precisely defined, whereas polysaccharides are of imprecisely known composition and molecular masses. Oligosaccharide‐to‐oligosaccharide transglycosylation with mannan hexasaccharides is shown in Figure 4C, Figure 9A and Figure 6B,D. To further explore how *Chara* enzymes can catalyse homo and hetero‐transglycosylation on oligosaccharides, we prepared diverse reaction mixtures with non‐radioactive Man_6_, Xyl_6_ or xyloglucan oligosaccharides and radioactive Man_6_‐ol, Xyl_6_‐ol and XXXGol (Figure 6). Products longer than the starting material, as judged by lower mobility on TLC, must indicate the occurrence of transglycosylation; smaller products are the result of hydrolysis and/or transglycosylation reactions. These experiments potentially reveal homo‐ and hetero‐activities of transglycosidase and transglycanase (exo‐ and endo‐enzymes, respectively) — which are dealt with individually below.

For better interpretation of activities detected in *Chara* crude extracts, consider first the radioactive products formed from a trace of [^3^H]Man_6_‐ol (~0.1 μM) in the presence of a higher concentration (~3 mM) of a qualitatively different non‐radioactive oligosaccharide, Xyl_6_ (Figure 6A, lanes 1 and 3).

#### 3.6.1 Mannan hydrolases

The [^3^H]Man_6_‐ol had been largely hydrolysed within 24 h, the major products being [^3^H]Man_3_‐ol > [^3^H]Man_4_‐ol; [^3^H]Man_5_‐ol and [^3^H]Man_2_‐ol were undetectable. This indicates that the principal reactions occurring were

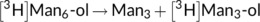

and some

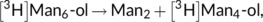

both catalysed by (endo) β‐mannanase acting near the middle of the hexasaccharide, but with negligible

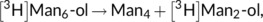

catalysed by β‐mannanase acting near the reducing terminus, nor

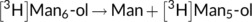

catalysed by (exo) β‐mannosidase.

Judged by the ^3^H‐labelled hydrolysis products observed, concentrated Xyl_6_ did not compete with the hydrolysis of [^3^H]Man_6_‐ol, suggesting the presence of a mannan‐specific hydrolase.

#### 3.6.2 Lack of xylan:mannan hetero‐transglycosylases

No radioactive products larger than [^3^H]Man_6_‐ol were detected with Xyl_6_ as the potential donor (Figure 6A, lanes 1 and 3), indicating negligible X:M transglycosylase actions such as

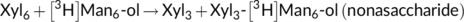

catalysed by a hypothetical heterotransxylanase, or

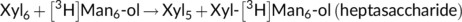

catalysed by a hypothetical heterotransxylosidase.

#### 3.6.3 Xylan hydrolases

In the case of tracer [^3^H]Xyl_6_‐ol plus (non‐interfering) non‐radioactive Man_6_, after 24 h some [^3^H]Xyl_6_‐ol remained intact but the majority had been hydrolysed to [^3^H]Xyl_5_‐ol, with smaller yields of [^3^H]Xyl_4_‐ol and a trace of [^3^H]Xyl_3_‐ol (Figure 6A, lanes 2 and 4), suggesting a progressive exo‐hydrolysis by β‐xylosidase activity:

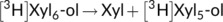




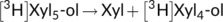




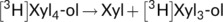




There was no evidence for a hypothetical β‐xylanase endo‐activity, which would have given [^3^H]Xyl_3_‐ol as the major product. Judged by the ^3^H‐labelled hydrolysis products observed, concentrated Man_6_ did not compete with the hydrolysis of [^3^H]Xyl_6_‐ol, suggesting the presence of a xylan‐specific hydrolase.

#### 3.6.4 Lack of mannan:xylan hetero‐transglycosylases

No radioactive products larger than [^3^H]Xyl_6_‐ol were detected, indicating negligible M:X transglycosylase action, either endo‐, e.g.

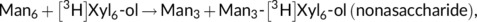

or exo‐, i.e.

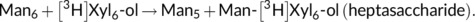




#### 3.6.5 Homo‐trans‐β‐mannosidase and homo‐trans‐β‐mannanase

Next consider the thymol‐stained (bulk) oligosaccharides in the reaction mixtures described above (Figure 6D). Even at 0.25 h, the Man_6_ contained traces of Man_3–5_ and bigger products (possibly Man_~9_) (lane 5); however, compared with these, large amounts of Man_2–5_ and Man_7–10_ and a chromatographically immobile polysaccharide had formed by 24 h (Figure 6D, lane 1). Clearly, Man_6_‐to‐Man_6_ homo‐transglycosylation was occurring, creating products bigger than the hexasaccharide (and necessarily accompanied by smaller products). The presence of abundant Man_7_ suggests trans‐β‐mannosidase action and the production of polysaccharide suggests additional transmannanase action. No free Man was generated, indicating that the transmannosidase activity was a dedicated transglycosylase possessing no detectable mannosidase (exo‐hydrolase) activity.

#### 3.6.6 Homo‐trans‐β‐xylanase but no detectable homo‐trans‐β‐xylosidase

At 0.25 h, the commercial Xyl_6_ was contaminated with traces of Xyl_5_ and Xyl_7_; however, against this background, it was clear that moderate amounts of Xyl_4_ and Xyl_3_ as well as Xyl_8–10_ (though negligible immobile polysaccharide) had formed. Thus again, transglycosylation was occurring, creating products bigger than the hexasaccharide (and accompanied by smaller products; Figures 6D line 2 and 5C line 1). The production of Xyl_8–10_ suggests (endo) transxylanase, catalysing reactions such as

$$2\;{\mathrm{Xyl}}_{6}\to {\mathrm{Xyl}}_{9}+{\mathrm{Xyl}}_{3}.$$

There was no detectable increase in Xyl_7_ between 0.25 and 24 h (Figure 6D, lanes 6 and 2), indicating negligible trans‐β‐xylosidase activity.

#### 3.6.7 Alternative method for detecting homo‐trans‐β‐mannosidase and homo‐trans‐β‐mannanase

Figure 6B shows experiments looking for homo‐transglycosylase activities as a comparison with Figure 6D but allowing us to trace specifically the reducing terminal [^3^H]alditol moiety. The reaction mixture [^3^H]Man_6_‐ol plus non‐radioactive Man_6_ is shown in lanes 1 and 5. As before, no radioactive products smaller than [^3^H]Man_3_‐ol were detected, but products larger than the hexasaccharide substrate were found, which appeared to be [^3^H]Man_7–10_‐ol and a [^3^H]polysaccharide. The formation of [^3^H]Man_5_‐ol in the presence of non‐radioactive Man_6_ supports the existence of a homo‐trans‐mannosidase that can transfer a single Man residue from [^3^H]Man_6_‐ol to Man_6_


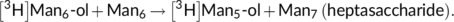




The formation of larger products e.g. [^3^H]Man_8–10_‐ol and even [^3^H]polysaccharides (designated as ‘[^3^H]Man_∞_‐ol’) indicates homo‐trans‐mannanase activity, catalysing, for example,

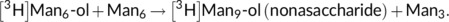




Repeated several times, this would generate a radioactive polymer, a process which might be rendered irreversible by the polysaccharide coming out of solution. Considering the bulk (thymol‐stained) sugars, we found that, as expected, Man_6_ in the presence of a trace of [^3^H]Man_6_‐ol (Figure 6D track 1) behaved exactly as Man_6_ in the presence of a trace of [^3^H]Xyl_6_‐ol (Figure 6C track 2; discussed above).

#### 3.6.8 Alternative method for seeking homo‐trans‐β‐xylosidase (not found) and homo‐trans‐β‐xylanase (found)

Hydrolysis of [^3^H]Xyl_6_‐ol was almost undetectable when non‐radioactive Xyl_6_ was also present (Figure 6B, lanes 2 and 6), indicating that the β‐xylosidase action on [^3^H]Xyl_6_‐ol (detected in Figure 6A, lane 2) was out‐competed by the ~3 mM Xyl_6_, which must therefore have been a concentration well above the *K*_M_. Nevertheless, a detectable proportion of the non‐radioactive Xyl_6_ was converted to Xyl_8–10_ (especially Xyl_9_), accompanied by Xyl_4_ and Xyl_3_ (Figure 6D, lane 2 compared with lane 6). It is difficult to judge the possible production of Xyl_7_ (which would suggest trans‐β‐xylosidase activity) since the hexasaccharide substrate was already slightly contaminated by the heptasaccharide. However, the yield of Xyl_9_ exceeded that of Xyl_8_, so it appears that a trans‐β‐xylanase predominated, catalysing the endo‐transglycosylation reaction

$$2\;{\mathrm{Xyl}}_{6}\to {\mathrm{Xyl}}_{9}+{\mathrm{Xyl}}_{3}$$

The possible production of Xyl_9_ by a hypothetical progressive trans‐β‐xylosidase (exo) activity, with the product of each step being the substrate for the next:

$$2\;{\mathrm{Xyl}}_{6}\to {\mathrm{Xyl}}_{7}+{\mathrm{Xyl}}_{5}$$

$\text{followed}\;\mathrm{by}:{\mathrm{Xyl}}_{7}+{\mathrm{Xyl}}_{6}\to {\mathrm{Xyl}}_{8}+{\mathrm{Xyl}}_{5}$

$$\text{and finally some}:{\mathrm{Xyl}}_{8}+{\mathrm{Xyl}}_{6}\to {\mathrm{Xyl}}_{9}+{\mathrm{Xyl}}_{5}$$

would have produced more Xyl_8_ than Xyl_9_.

#### 3.6.9 Lack of xyloglucan‐acting α‐xylosidase, trans‐α‐xylosidase, β‐galactosidase and trans‐β‐galactosidase

To look for (exo) enzyme activities that might act on xyloglucan oligosaccharides, we monitored the action of *Chara* enzymes on a trace (~0.1 μM) of [^3^H]XXXGol in the presence higher concentrations (~3 mM) of non‐radioactive xyloglucan oligosaccharides. The non‐radioactive oligomers tested were a relatively pure heptasaccharide (XXXG) and a mixture of nona‐ to heptasaccharides (mainly XLLG > XXLG > XXXG). Figure 6B (lanes 3,4,7 and 8) shows no reaction of the radioactive substrate (which could have been a donor or acceptor). Furthermore, Figure 6D (same lanes) shows no hydrolysis or transglycosylation of the non‐radioactive substrates. Thus, the *Chara* extract contained no detectable α‐xylosidase, trans‐α‐xylosidase, β‐galactosidase or trans‐β‐galactosidase. It was not expected that endo‐enzyme activities capable of acting within xyloglucan oligosaccharides would be present as these are not known from land plants and because *Chara* lacks biochemically detectable xyloglucan.

### 3.7 Kinetics of trans‐β‐mannosidase and trans‐β‐mannanase activities from *Chara*

As trans‐β‐mannosidase and trans‐β‐mannanase were major wall‐polymer remodelling activities detected in *Chara* extracts (Figure 6B,C,D), we examined the kinetics of these enzymes. We tested 0–3027 μM Man_6_ as the donor and 0.1 μM [^3^H]Man_6_‐ol as acceptor, which allowed us to track the formation of the transglycosylation products (Figure S4). The radioactive products were separated by TLC and quantitatively scanned. After 24 h incubation, the exo‐transglycosylation product [^3^H]Man_7_‐ol was detectable at the relatively low donor substrate concentration of 101 μM (Figure 7B and 8D). With higher donor concentrations (≥ 505 μM), the 24‐h yield of [^3^H]Man_7_‐ol had peaked, and there was little difference between 505 and 3027 μM donor.

Higher‐homologue products ([^3^H]Man_8–10_‐ol and [^3^H]polysaccharides), indicative of trans‐β‐mannanase activity, were also clearly visible when the donor was ≥505 μM, though not at 101 μM (Figure 7B and D). These findings suggest that trans‐β‐mannosidase has a lower *K*_M_ for Man_6_ (donor) and is a more ‘dedicated’ transglycosylase activity than trans‐β‐mannanase.

Keeping the donor concentration constant at 1000 μM, we tracked the 0–24‐h time‐course of formation of transglycosylation products (Figure 7A and C). The [^3^H]Man_9_‐ol and [^3^H]polysaccharide products (indicative of endo‐transglycosylation) appeared first, becoming detectable within 10–30 min. The yield of [^3^H]Man_9_‐ol plateaued at ~1 h, and thereafter remained steady for at least 24 h, whereas the yield of [^3^H]polysaccharide continued to rise for at least 12 h. This suggests that [^3^H]Man_9_‐ol was an intermediate, en route to the production of (relatively insoluble and thus stable) [^3^H]polysaccharides:

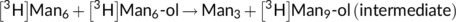




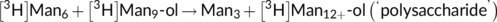




The [^3^H]Man_7_‐ol product (indicative of exo‐transglycosylation) appeared more gradually, continuing to rise up to at least 24 h (Figure 7A,C). The results thus indicate that the endo‐enzyme trans‐β‐mannanase, despite having a lower affinity (higher *K*_M_) for Man_6_, was ultimately more active than endo‐enzyme trans‐β‐mannosidase.

### 3.8 Transglycanase activity utilising oligomannan as acceptor substrate detected *in situ* acting on endogenous wall polysaccharides

**FIGURE 10:**
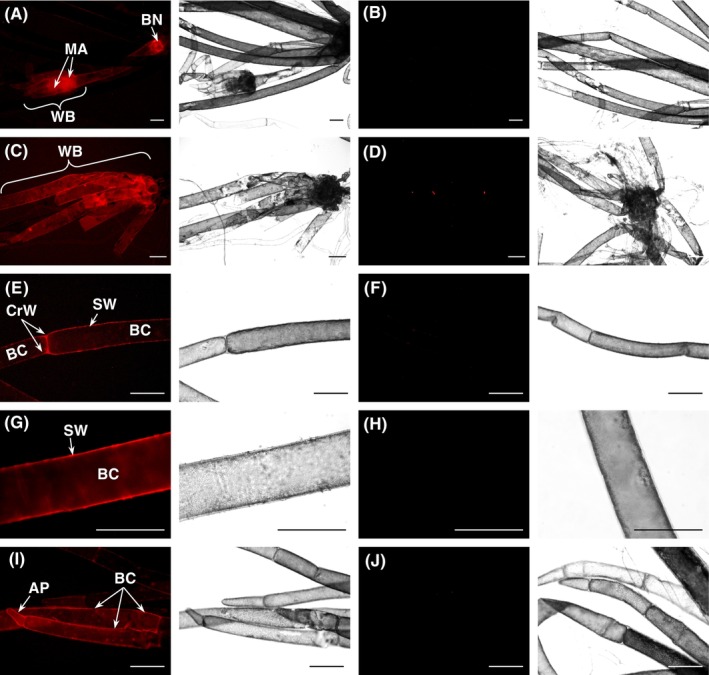
*In‐situ* action of trans‐glycanase(s) on native endogenous polysaccharides of *Chara vulgaris*.Fluorescence and corresponding bright‐field micrographs of specimens fed with 5 μM fluorescently tagged Man_6_‐ol–SR, to which *Chara* cell‐wall polysaccharides became grafted. (A) Transglycanase action in the basal node (BN) and multicellular meristematic apex (MA) of axillary whorl of ‘talon’‐like branchlets (WB). (C) The third whorl of branchlets of the main axis. (E) Fluorescent side walls (SW) and cross‐wall (CrW) between two adjacent branchlet cells (BC). (G) A detail of Man_6_‐ol–SR‐tagged side cell wall (SW) of elongated branchlet cell. (I) A detail of a fluorescent branchlet apex (AP) and branchlet cells (BC). Controls (B,D,F,H,J) show pre‐boiled *Chara* (thus containing heat‐denatured transglycanases) incubated with 5 μM Man_6_‐ol–SR. Scale bar = 250 μm.

An *in‐situ* approach performed by fluorescence microscopy (Figure 10) revealed that Man_6_‐ol–SR can serve as acceptor substrate for endogenous transglycanase(s) acting on intrinsic donor polysaccharides in *Chara* cell walls. Fluorescent signals were detected in the multicellular meristematic apex of axillary whorls, in the side‐ and cross‐wall between two adjacent branchlets and especially in the branchlet cells. Judged by the fluorescent signal from wall‐incorporated Man_6_‐ol–SR, the detected transglycanase(s) appear to ‘cut and paste’ wall polysaccharide(s), with oligomannan as acceptor substrate, in most *Chara* cells.

## 4 DISCUSSION

### 4.1 Setting the scene

Several land‐plant transglycanase and transglycosidase activities have been detected, which certainly *in vitro* and in some cases demonstrated *in vivo*, are capable of re‐structuring the major cell‐wall polysaccharides of land‐plants. Many of those activities are found throughout the (land‐) plant kingdom, though some are confined to (or only prominent in) certain taxa such as trans‐β‐mannanase in some ferns, hornworts and lycophytes (Franková & Fry, 2021), and MXE and CXE in *Equisetum* (Simmons et al., 2015; Herburger et al., 2021). In this work, we extended the search for such enzymes to a charophytic alga, *Chara vulgaris*. *Chara* is a late‐diverging (formerly termed ‘higher’) charophyte, i.e., relatively closely related to land plants. Indeed, *Chara* shares several cell‐wall polysaccharides with land‐plants, but those from *Chara* are not fully characterised, especially their side chains and linkages; therefore, we used land‐plant polysaccharides as substrates in our assays.

### 4.2 XET‐like and MXE‐like activities detected

Transglycanase (i.e., endo‐) activities that use a xyloglucan‐related acceptor substrate ([^3^H]XXXGol) were readily detected in *Chara* extracts (Figures 3A and 2). These enzymes were able to utilise xyloglucan and MLG as donor substrate (i.e., they exhibited XET and MXE activities), cutting the donor in mid‐chain and grafting a portion of it onto the XXXGol. XET activity is well known throughout the land‐plants, and MXE activity is found in *Equisetum*. It is surprising to find these two activities in *Chara* since this alga possesses no biochemically detectable xyloglucan or MLG. Acid hydrolysis of *Chara* hemicellulose does yield (mainly) Glc, Xyl and Man (O'Rourke et al., 2015), so we suggest that *Chara* may have a hemicellulose that resembles xyloglucan or MLG; however, this hypothetical polysaccharide must differ in some chemical feature that renders it resistant to enzymes routinely employed in land‐plant hemicellulose analysis (namely Driselase, yielding isoprimeverose; XEH, yielding XGOs; and lichenase, yielding a trisaccharide plus a tetrasaccharide) (Popper & Fry, 2003). Further work on *Chara* hemicelluloses is required to identify the native substrate of the ‘XET‐like’ and ‘MXE‐like’ activities detected here. Nevertheless, our conclusion is that *Chara* shares with the land‐plants certain fundamental enzymic tools involved in cell‐wall restructuring. These tools were also found in other representatives of charophytes — *Nitella, Klebsormidium, Spirogyra, Zygnema* and *Coleochaete* (Franková & Fry, 2021; Herburger et al., 2018). However, it was mainly Charophyceae (*Chara* and *Nitella* sp.) exhibiting high XET‐ and MXE‐like activities (Franková & Fry, 2021).

*Chara* does possess cellulose, the donor substrate for *Equisetum* CXE activity. However, there was no evidence of a ‘CXE’ activity capable of acting on the model donor substrate, water‐soluble cellulose acetate. But this may not be surprising since *Equisetum* HTG has low ‘CXE’ activity on water‐soluble cellulose acetate (Simmons et al., 2015) but prefers unmodified cellulose type II and I (Herburger et al., 2020b) .

*Chara* extracts did not exhibit either of the transglycosidase (i.e., exo‐) activities assayed — trans‐α‐xylosidase and trans‐β‐galactosidase — when xyloglucan‐derived oligosaccharides were tested as potential substrates. This tallies with the consensus that *Chara* lacks land‐plant‐like xyloglucan and the enzymes specifically adapted to restructure it.

### 4.3 Transglycanases and transglycosylases acting on mannans

(1→4)‐β‐d‐Mannans are major hemicelluloses of the *Chara* cell wall, so it seemed more likely that trans‐β‐mannanases and trans‐β‐mannosidases might be present to allow their restructuring *in vivo*. Indeed, both these activities were readily detectable *in vitro* in *Chara* extracts (summarised in Figure 2). Routinely, we used [^3^H]Man_6_‐ol as the acceptor substrate, representing a fragment of the backbone of mannan. Trans‐β‐mannanase activity was detected with any of a range of donor substrates: pure (1→4)‐β‐d‐mannan, glucomannan, galactomannan and mannohexaose. We suggest that this enzyme activity serves the role of ‘cutting and pasting’ chains of the major hemicellulose, mannan, in the *Chara* cell wall, enabling cell expansion in the manner previously proposed for XET activity (on xyloglucan) in land‐plants (Thompson & Fry, 2001; Hayashi & Kaida, 2011; Franková & Fry, 2013).

Interestingly, we also detected a hetero‐trans‐β‐mannanase activity capable of using galactomannan as donor substrate when the acceptor substrate was a fragment ([^3^H]Xyl_6_‐ol) of qualitatively different hemicellulose, (1→4)‐β‐d‐xylan. If this enzyme acts *in vivo*, it can be envisaged that *Chara* is able to graft portions of mannan onto the non‐reducing terminus of a xylan, creating a mannan–xylan hybrid polysaccharide. We are not in a position to judge what the mechanical consequences of such grafting might be since the distinct roles of mannan versus xylan in wall architecture are not known. Since mannans are abundant hemicelluloses in many charophytes (Domozych & LoRicco, 2023; O'Rourke et al., 2015; Permann et al., 2021), it is likely that mannan‐to‐xylan enzymic crosslinking may bring structural strength in *Chara* primary cell wall similarly to enzymic crosslinking provided by HTG in *Equisetum*, where maturing tissues appeared to undergo MLG–xyloglucan and cellulose–xyloglucan grafting (Simmons et al., 2015, Herburger et al., 2020a).

The exo‐enzyme activity, homo‐trans‐β‐mannosidase, was also detected (Figure 6, Figure 7, Figure 2). This was demonstrated by the use of the hexasaccharide fragment, Man_6_, as donor substrate and either a second Man_6_ molecule or [^3^H]Man_6_‐ol as acceptor. Since this activity attacks the donor substrate specifically at the non‐reducing terminus of the chain, it is more easily detected acting on a hexasaccharide than on a polysaccharide (in which very few termini are present relative to the total mass). It is unclear what the role of this exo‐activity might be — slightly ‘nibbling’ the substrate from the end rather than ‘biting’ it in the middle. But, in general terms, it is possible that tweaking the non‐reducing end could render that terminus a better or worse acceptor substrate for mannan‐to‐mannan endo‐transglycosylation. For example, by removing the terminal β‐Man residue it could leave the polysaccharide with a substituted Man residue (e.g. one with a galactosyl or acetyl side‐chain) at the terminus, which might be a poor acceptor substrate for trans‐β‐mannanase; this would take that polysaccharide molecule ‘out of the game’ (at least as an acceptor substrate) for wall re‐structuring.

Trans‐glycanase action was also detected on the intrinsic cell wall components of *Chara*, with exogenous fluorescent Man_6_‐ol–SR as the acceptor substrate and endogenous polysaccharide(s) as donor (Figure 10). Potentially, the observed activity was TBM, though the possibility cannot be excluded that the *in‐situ* donor substrate was not a mannan — for example, the activity could be xylan:oligomannan hetero‐transxylanase, though this is unlikely because no such activity was detectable in enzyme extracts *in vitro* (Figure 2B).

Man_6_‐ol–SR as acceptor substrate was also used in the dot‐blot method (Figure S3), which demonstrated *Chara* TBM's *in‐vitro* activity on glucomannan. Despite both *Equisetum* and *Chara* cell walls being rich in Man residues (Nothnagel & Nothnagel, 2007; Silva et al., 2011), only *Chara* extracts formed bright fluorescent spots on the test papers. This indicates mannan‐remodelling is more favoured in charophytes than in the Equisetales.

### 4.4 Transglycanases and transglycosidases acting on xylans

(1→4)‐β‐d‐Xylans also appear to be abundant in *Chara* cell walls (O'Rourke et al., 2015; Popper & Fry, 2003; Nishiyama et al., 2018) and therefore we looked for trans‐β‐xylanase and trans‐β‐xylosidase activities. Indeed, trans‐β‐xylanase was demonstrated using xylan or arabinoxylan as donor substrate and [^3^H]Xyl_6_‐ol as acceptor (Figure 3C). It was also detectable with Xyl_6_ as both donor and acceptor. Thus, we can propose that *Chara* is capable of re‐structuring its xylan‐type hemicelluloses, with likely consequences for wall properties and cell growth.

No hetero‐trans‐β‐xylanase activity was detected with xylans as donor and [^3^H]Man_6_‐ol as acceptor. Thus, we did not detect an enzyme capable of creating xylan–mannan hybrid polysaccharides. We conclude that *Chara* can potentially make mannan→xylan hybrid hemicelluloses (where ‘→’ is a glycosidic bond) but not xylan→mannan hybrids. The significance of this distinction concerning charophyte wall architecture remains to be elucidated.

The exo‐enzyme, homo‐trans‐β‐xylosidase, was not detected. Thus *Chara* appears unable to ‘nibble’ xylans in the manner proposed above for mannans.

### 4.5 Dedicated *versus* mechanistic transglycosylases: crucial importance of acceptor substrate concentration

There is little fundamental difference between a transglycosylase activity and a hydrolase activity (Franková & Fry, 2013). The latter can be regarded as a special case of the former in which the acceptor substrate is H_2_O rather than an organic molecule such as a carbohydrate. Indeed, many hydrolases can catalyse transglycosylation reactions *in vitro* if a high enough concentration of carbohydrate is present. On the other hand, some enzymes are ‘dedicated’ transglycosylases, such as most XET‐active enzymes (Shi et al., 2015), unable to catalyse appreciable rates of hydrolysis. On the spectrum between ‘dedicated’ transglycosylases and predominant hydrolases, the type of reaction catalysed *in vivo* will depend on the concentration of potential acceptor substrate molecules (relative to H_2_O, which is always ~55 M). The concentration of the donor (or hydrolysable) substrate will have little influence on the transglycosylation versus hydrolysis balance (except that the intended ‘donor’ substance may also act as an acceptor if concentrated enough).

In many of our experiments, the acceptor leading to detectable transglycosylation was a radiolabelled oligosaccharide present in the order of 0.1–2.0 μM concentrations. Since we detected transglycosylation under those conditions, we conclude that the observed transglycanase or transglycosylase was capable of catalysing transglycosylation at vanishing low acceptor concentrations and was thus not purely a hydrolase. Therefore, the enzyme is likely to be capable of catalysing transglycosylation *in vivo* at substrate concentrations naturally occurring in the cell wall.

Indeed, the reaction rate in trans‐β‐mannanase assays appeared to plateau above about 0.3–0.4 μM [^3^H]Man_6_‐ol (acceptor substrate) concentration, suggesting that the *K*_M_ of the enzymes was at or below this range. This observation indicates a very high affinity (low *K*_M_) of the enzyme for non‐reducing terminal Man residues, again strongly supporting the view that the enzymic reaction (trans‐β‐mannanase) can operate in the cellular environment.

Since the acceptor substrate site is assumed to be the non‐reducing terminus of the hemicellulose backbone (i.e., one site per polysaccharide molecule), it is relevant to consider the molar concentration of the acceptor substrate rather than its w/v concentration. Calculating the *in‐vivo* molarity of a hemicellulose depends on the w/v concentration of the polysaccharide in the cell wall and its relative molecular mass (M_r_). As an order of magnitude, the value of 1 μM mentioned above seems reasonable. Therefore, we conclude that the enzymes detected in this work by use of radiolabelled acceptor substrates would be capable of operating *in vivo*.

In other experiments, we used non‐radioactive oligosaccharides as both donor and acceptor. In these cases, the oligosaccharide (thus acceptor substrate but also, irrelevantly, the donor) concentration was 0–3 mM. The lowest concentration showing clear‐cut transglycosylation under these conditions was ~100 μM, which we suggest is also about within the range of polysaccharide concentrations occurring in the cell‐wall matrix, depending on M_r_. Thus, again, the data support the proposed ability of the detected enzymes to re‐structure *Chara* hemicelluloses *in vivo*.

### 4.6 Dependence of trans‐glycanase and trans‐glycosidase action on donor substrate concentrations

The concentration‐dependence of the donor substrate is less relevant to understanding the main role (hydrolysis versus transglycosylation) of these enzymes. However, we can record that when mannan, glucomannan, Man_6_ and xylan were used as donor, the transglycosylation rate reached a plateau at roughly 1 mg/mL, and above that did not increase. Since the donor substrate can be attacked by the transglycanases at essentially any appropriate glycosidic linkage along the backbone chain, the more relevant units to quote are w/v concentration rather than molarity. The donors tested had a wide range of M_r_ values, thus the common figure of ~1 mg ml^−1^ (w/v; Figure 4) corresponds to a wide range of molarities (~2 μM glucomannan, ~400 μM for pure β‐1,4‐mannan, 1000 μM Man_6_, and ~ 10 μM xylan. It is clear from this that, as expected, the donor molarity is largely irrelevant, and that the 1 mg ml^−1^ concentration found to give maximal rates is what matters. A polysaccharide concentration of ~1 mg ml^−1^ in the cell‐wall matrix *in vivo* is low compared with realistic estimates of typical cell‐wall compositions (hemicellulose:H_2_O ratio; Monro et al., 1976; 20% hemicellulose of total mass present in the primary cell wall, Varner & Lin, 1989).

### 4.7 The isoforms of *Chara* transglycanases

IEF showed that at least three separate enzymes were present:

1. with XET activity (pI 7.4)
2. with XET and MXE activities (pI 4.3)
3. two with TBM activity (pI 4.3 and 7.0).

We cannot rule out the possibility that the two pI‐4.3 activities are catalysed by a single protein with a relatively lax donor substrate specificity. Future work will be required to test whether these enzymes are themselves multiple and whether they have homology with related land‐plant enzymes.

## 5 CONCLUSIONS

This is the first study to explore in depth the transglycanase and transglycosidase (endo‐ and exo‐, respectively) activities likely to act on the cell walls of *Chara*, a ‘late‐diverging’ charophytic alga relatively closely related to the land‐plants. We show that *Chara* possesses activities capable of re‐structuring two of its major hemicelluloses: mannan‐based (by endo‐ and exo‐transglycosylation) and xylan‐based (by endo‐transglycosylation). There was also evidence for mannan‐to‐xylan hetero‐transglycosylation (but not xylan‐to‐mannan transglycosylation), thus providing *Chara* with the capacity to produce mannan→xylan ‘hybrid’ polysaccharides (Figure 2). More surprisingly, we found enzyme activities that can catalyse xyloglucan‐to‐xyloglucan endo‐transglycosylation and MLG‐to‐xyloglucan endo‐transglycosylation, suggesting that *Chara* possesses hemicelluloses functionally resembling xyloglucan and/or MLG, but differing in sufficient structural features that these polymers cannot be detected by chemical analysis using techniques which very sensitively reveal xyloglucan and MLG in land‐plant cell walls. Evidence for traces of epitopes detected by xyloglucan‐ and MLG‐‘specific’ antibodies (Mikkelsen et al., 2021) is not conclusive because the precise range of epitope(s) recognised by these antibodies can never be comprehensively defined. Results entirely based on antibodies are not always reliable, as reported by Pfeifer et al. (2023), who found that antibodies raised against arabinogalactan‐protein (AGP) epitopes cross‐reacted with unconventional methylated galactan in four *Chara* species that lack AGPs. Future work will be required to demonstrate to what extent *Chara* possesses xyloglucan‐ and MLG‐like hemicelluloses. *Chara* certainly did not exhibit any trans‐glycosidase activities capable of acting on fragments of land‐plant xyloglucan. Based on the predominant detected homo‐ and hetero‐ TBM > TBX and XET/MXE‐like activities, we assume that mannan‐, xylan‐ and xyloglucan‐like polysaccharide‐remodelling is well represented in *Chara* and thus may have originated prior to the divergence of the Charophyceae from their sister clade (Li et al., 2020) which contains the Zygnematophyceae, Coleochaetophyceae and land‐plants.

In conclusion, we present evidence that *Chara* has a sophisticated arsenal of enzymic ‘tools’ capable of re‐structuring its cell‐wall matrix, thereby potentially contributing to the mechanism and regulation of cell expansion, which may represent the evolutionary origins of the wall hemicellulose re‐structuring mechanisms much studied in land‐plants.

## AUTHOR CONTRIBUTIONS

Both SCF and LF planned and designed the study, LF performed the experiments, prepared the figures and processed the data. Both LF and SCF drafted and edited the manuscript.

## CONFLICT OF INTEREST STATEMENT

Both the authors declare that they have no competing interest that could have appeared to influence the work reported in this document.

## Supporting information


**Figure S1.** Trans‐β‐mannanase vs. hydrolase activities in extracts from *Chara vulgaris* and *Nitella flexilis*
**Figure S2.** Testing the optimal oligomannan concentration for trans‐β‐mannanase from *Chara vulgaris*
**Figure S3.** Dot‐blot assay for trans‐β‐mannanase activity extracted from *Chara vulgaris* and *Equisetum* spp.
**Figure S4.** Controls for kinetic properties of oligomannan‐remodelling transglycosylases extracted from *Chara vulgaris*

## Data Availability

Not applicable; all new created data are contained within this article and supplementary document.
