# Supplementary material for: Chara — a living sister to the land plants with pivotal enzymic toolkit for mannan and xylan remodelling
Source: Physiol Plant. 2023 Dec 29;176(1):e14134. doi: 10.1111/ppl.14134 (PMC10962555; doi:10.1111/ppl.14134)
Supplement: Supplementary file 1 — Figure S1. Trans‐β‐mannanase vs. hydrolase activities in extracts from Chara vulgaris and Nitella flexilis Figure S2. Testing the optimal oligomannan concentration for trans‐β‐mannanase from Chara vulgaris Figure S3. Dot‐blot assay for trans‐β‐mannanase activity extracted from Chara vulgaris and Equisetum spp. Figure S4. Controls for kinetic properties of oligomannan‐remodelling transglycosylases extracted from Chara vulgaris [file PPL-176-0-s001.pptx]

## Slide 1
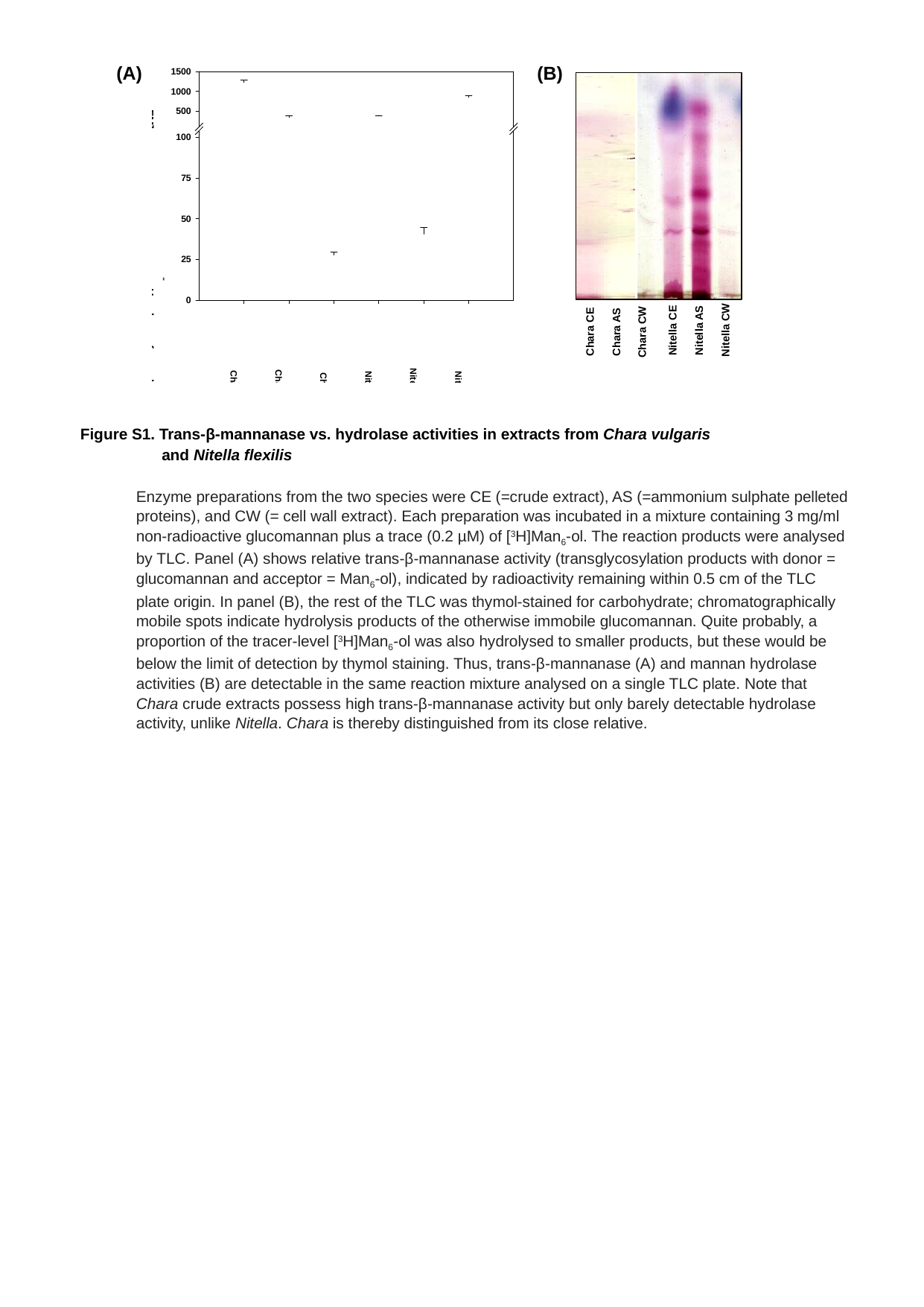

(A)
(B)
-
Nitella CW
Nitella CE
Nitella AS
Chara CE
Chara AS
Chara CW
Figure S1. Trans-β-mannanase vs. hydrolase activities in extracts from Chara vulgaris
 and Nitella flexilis
Enzyme preparations from the two species were CE (=crude extract), AS (=ammonium sulphate pelleted proteins), and CW (= cell wall extract). Each preparation was incubated in a mixture containing 3 mg/ml non-radioactive glucomannan plus a trace (0.2 µM) of [3H]Man6-ol. The reaction products were analysed by TLC. Panel (A) shows relative trans-β-mannanase activity (transglycosylation products with donor = glucomannan and acceptor = Man6-ol), indicated by radioactivity remaining within 0.5 cm of the TLC plate origin. In panel (B), the rest of the TLC was thymol-stained for carbohydrate; chromatographically mobile spots indicate hydrolysis products of the otherwise immobile glucomannan. Quite probably, a proportion of the tracer-level [3H]Man6-ol was also hydrolysed to smaller products, but these would be below the limit of detection by thymol staining. Thus, trans-β-mannanase (A) and mannan hydrolase activities (B) are detectable in the same reaction mixture analysed on a single TLC plate. Note that Chara crude extracts possess high trans-β-mannanase activity but only barely detectable hydrolase activity, unlike Nitella. Chara is thereby distinguished from its close relative.

## Slide 2
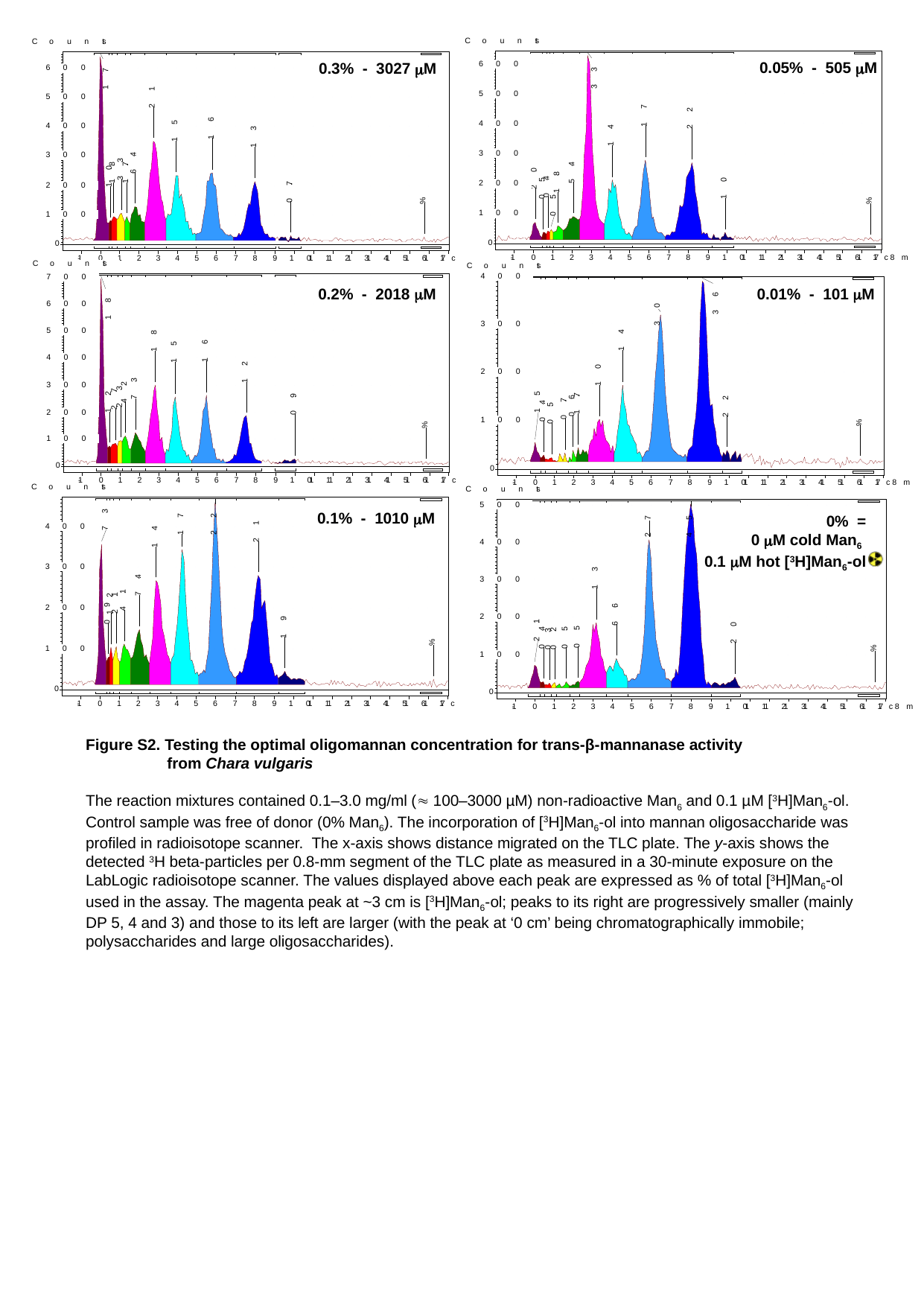

0.05% - 505 mM
0.3% - 3027 mM
0.2% - 2018 mM
0.01% - 101 mM
0.1% - 1010 mM
0% =
 0 mM cold Man6
0.1 mM hot [3H]Man6-ol
Figure S2. Testing the optimal oligomannan concentration for trans-β-mannanase activity
 from Chara vulgaris
The reaction mixtures contained 0.1–3.0 mg/ml ( 100–3000 µM) non-radioactive Man6 and 0.1 µM [3H]Man6-ol. Control sample was free of donor (0% Man6). The incorporation of [3H]Man6-ol into mannan oligosaccharide was profiled in radioisotope scanner. The x-axis shows distance migrated on the TLC plate. The y-axis shows the detected 3H beta-particles per 0.8-mm segment of the TLC plate as measured in a 30-minute exposure on the LabLogic radioisotope scanner. The values displayed above each peak are expressed as % of total [3H]Man6-ol used in the assay. The magenta peak at ~3 cm is [3H]Man6-ol; peaks to its right are progressively smaller (mainly DP 5, 4 and 3) and those to its left are larger (with the peak at ‘0 cm’ being chromatographically immobile; polysaccharides and large oligosaccharides).

## Slide 3
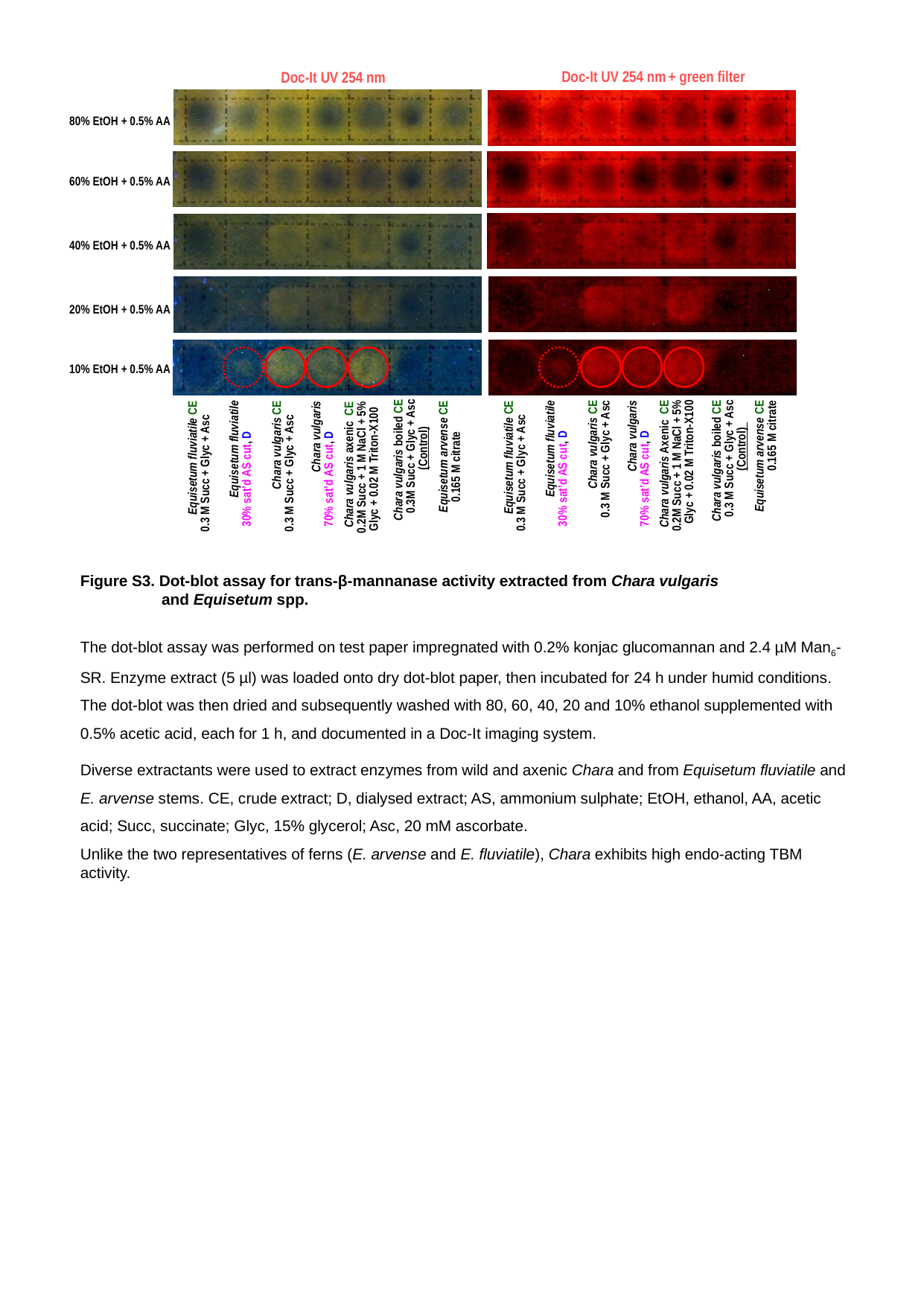

Doc-It UV 254 nm + green filter
Doc-It UV 254 nm
80% EtOH + 0.5% AA
60% EtOH + 0.5% AA
40% EtOH + 0.5% AA
20% EtOH + 0.5% AA
10% EtOH + 0.5% AA
Chara vulgaris Axenic CE
0.2M Succ + 1 M NaCl + 5% Glyc + 0.02 M Triton-X100
Chara vulgaris axenic CE
0.2M Succ + 1 M NaCl + 5% Glyc + 0.02 M Triton-X100
Chara vulgaris boiled CE
0.3M Succ + Glyc + Asc
(Control)
Chara vulgaris boiled CE
0.3 M Succ + Glyc + Asc
(Control)
Equisetum fluviatile CE
0.3 M Succ + Glyc + Asc
Equisetum fluviatile
30% sat’d AS cut, D
Chara vulgaris CE
0.3 M Succ + Glyc + Asc
Chara vulgaris
70% sat’d AS cut, D
Equisetum arvense CE
0.165 M citrate
Equisetum fluviatile CE
0.3 M Succ + Glyc + Asc
Equisetum fluviatile
30% sat’d AS cut, D
Chara vulgaris CE
0.3 M Succ + Glyc + Asc
Chara vulgaris
70% sat’d AS cut, D
Equisetum arvense CE
0.165 M citrate
Figure S3. Dot-blot assay for trans-β-mannanase activity extracted from Chara vulgaris
 and Equisetum spp.
The dot-blot assay was performed on test paper impregnated with 0.2% konjac glucomannan and 2.4 µM Man6-SR. Enzyme extract (5 µl) was loaded onto dry dot-blot paper, then incubated for 24 h under humid conditions. The dot-blot was then dried and subsequently washed with 80, 60, 40, 20 and 10% ethanol supplemented with 0.5% acetic acid, each for 1 h, and documented in a Doc-It imaging system.
Diverse extractants were used to extract enzymes from wild and axenic Chara and from Equisetum fluviatile and E. arvense stems. CE, crude extract; D, dialysed extract; AS, ammonium sulphate; EtOH, ethanol, AA, acetic acid; Succ, succinate; Glyc, 15% glycerol; Asc, 20 mM ascorbate.
Unlike the two representatives of ferns (E. arvense and E. fluviatile), Chara exhibits high endo-acting TBM activity.

## Slide 4
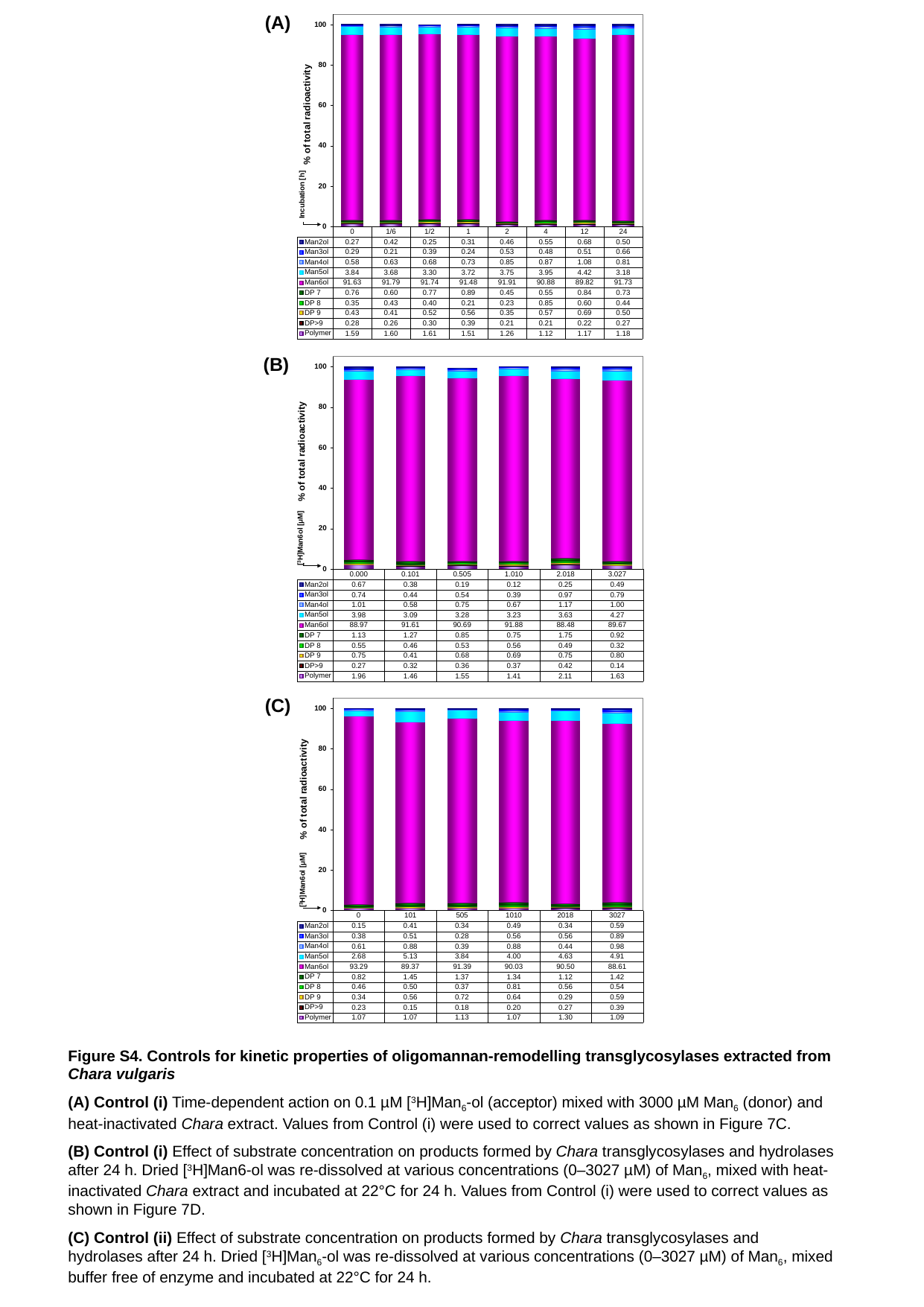

(A)
% of total radioactivity
(B)
% of total radioactivity
(C)
% of total radioactivity
Figure S4. Controls for kinetic properties of oligomannan-remodelling transglycosylases extracted from Chara vulgaris
(A) Control (i) Time-dependent action on 0.1 µM [3H]Man6-ol (acceptor) mixed with 3000 µM Man6 (donor) and heat-inactivated Chara extract. Values from Control (i) were used to correct values as shown in Figure 7C.
(B) Control (i) Effect of substrate concentration on products formed by Chara transglycosylases and hydrolases after 24 h. Dried [3H]Man6-ol was re-dissolved at various concentrations (0–3027 µM) of Man6, mixed with heat-inactivated Chara extract and incubated at 22°C for 24 h. Values from Control (i) were used to correct values as shown in Figure 7D.
(C) Control (ii) Effect of substrate concentration on products formed by Chara transglycosylases and hydrolases after 24 h. Dried [3H]Man6-ol was re-dissolved at various concentrations (0–3027 µM) of Man6, mixed buffer free of enzyme and incubated at 22°C for 24 h.
